# Intratumoral heterogeneity of intrahepatic cholangiocarcinoma

**DOI:** 10.18632/oncotarget.14844

**Published:** 2017-01-27

**Authors:** Dirk Walter, Claudia Döring, Magdalena Feldhahn, Florian Battke, Sylvia Hartmann, Ria Winkelmann, Markus Schneider, Katrin Bankov, Andreas Schnitzbauer, Stefan Zeuzem, Martin Leo Hansmann, Jan Peveling-Oberhag

**Affiliations:** ^1^ Department of Internal Medicine I, Johann Wolfgang Goethe-University Hospital, 60590 Frankfurt, Germany; ^2^ Dr. Senckenberg Institute of Pathology, Johann Wolfgang Goethe-University Hospital, 60590 Frankfurt, Germany; ^3^ CeGaT GmbH, 72076 Tuebingen, Germany; ^4^ Department of General and Visceral Surgery, Johann Wolfgang Goethe-University Hospital, 60590 Frankfurt, Germany; ^5^ Department for Gastroenterology, Hepatology and Endocrinology, Robert-Bosch Hospital, 70376 Stuttgart, Germany

**Keywords:** cholangiocarcinoma, exome sequencing, intratumoral heterogeneity, microsatellite instability, MSH6

## Abstract

No personalized therapy regimens could demonstrate a benefit in survival of intrahepatic cholangiocarcinoma (iCCA). Since genetic heterogeneity might influence single biopsy based targeted therapy or the outcome of clinical trials, aim of the present study was to investigate intratumoral heterogeneity of iCCA by whole exome sequencing. Therefore, samples from tumor center and tumor periphery of large iCCA lesions as well as a control from healthy liver tissue were obtained from four patients and whole exome sequencing was performed. Mutations that occurred only in the tumor center or periphery were defined as private, whereas mutations present in both samples were regarded as common. A mean of 3 non-synonymous private mutations (range 0–14) per sample compared to 33,3 common mutations per sample (range 24–41) was identified. Mean percentage of non-synonymous private mutations per sample was 12% (range 0–58). In all samples of patient 1-3 as well as the central sample of patient 4 ≤ 10% private mutations were found, whereas 58% of private mutations were identified in the peripheral sample of patient 4. In this sample a private mutation in the DNA mismatch repair protein MSH6 could be identified most likely causing the high amount of private mutations. No substantial intratumoral heterogeneity was found in copy number variation analysis. In conclusion, iCCA show a small but distinct intratumoral heterogeneity. Somatic mutations in mismatch repair proteins might contribute significantly to increased spatial tumor burden and thereby may influence clinical management.

## INTRODUCTION

Cholangiocarcinoma (CCA) is a gastrointestinal neoplasia deriving from biliary epithelium or peribiliary glands. It is classified according to its anatomical origin as intrahepatic (iCCA), perihilar or distal cholangiocarcinoma, whereas intrahepatic is defined as proximally to the second degree bile ducts [1]. iCCA is the second leading cause for primary liver cancer and incidence varies between 2/100,000 in western countries and > 80/100,000 in Thailand [2]. Median survival for unresectable tumors (60–70% of all iCCA) is 12–15 months, while 5-year survival for resectable iCCA is 10–40% despite surgery, which is the only option of curative therapy [2]. Many approaches of personalized therapy regimens have been evaluated, but so far, none could demonstrate a benefit in survival compared to the current standard treatment consisting of a systemic chemotherapy with platinum and gemcitabine [3].

Reasons for the poor response rates of targeted therapy regimens in iCCA are intensively discussed. One reason might be a dense and hypovascularized desmoplastic stroma, a characteristic of iCCA which impedes interaction of cytotoxic and targeted drugs with neoplastic cells [4]. On the other hand, due to broader use of next generation sequencing, a profound genetic heterogeneity of CCA was discovered in recent years [5, 6]. This is of special interest since many trials with personalized therapy regimens were designed for all biliary tract neoplasms and thereby might have combined different mutational profiles.

Besides delineation of iCCA to extrahepatic CCA, recent research increasingly focused on genetic variability of iCCA. For example, two molecular subclasses of iCCA with different outcomes were identified with whole transcriptome analyses [7, 8]. Furthermore, different cells of origin are discussed such as biliary epithelial cells, hepatic progenitor cells or mature hepatocytes [9].

Besides variations in the genetic landscape between different subclasses of CCA (intertumoral heterogeneity), recent data of hepatocellular carcinoma suggested development of different subclones in primary liver tumors and intrahepatic satellite nodules of the same patient, which is known as intratumoral heterogeneity (ITH) [10]. Moreover, studies on lung adenocarcinoma and clear cell renal carcinoma could show that genetic subclones already exist in the primary lesion itself and that the extent of subclonality between tumor entities can be clearly different [11–13]. However, to date there is no data on ITH of iCCA and further investigation is an urgent need given the devastating prognosis these patients have.

In the current study, we performed whole exome sequencing of samples from tumor center and tumor periphery as well as corresponding healthy liver tissue of four patients with iCCA. By comparing the presence of somatic mutations in the matched tumor center and tumor periphery samples of each patient we determined the occurrence and extent of ITH in iCCA.

## RESULTS

### Sequencing data

**Figure 1:**
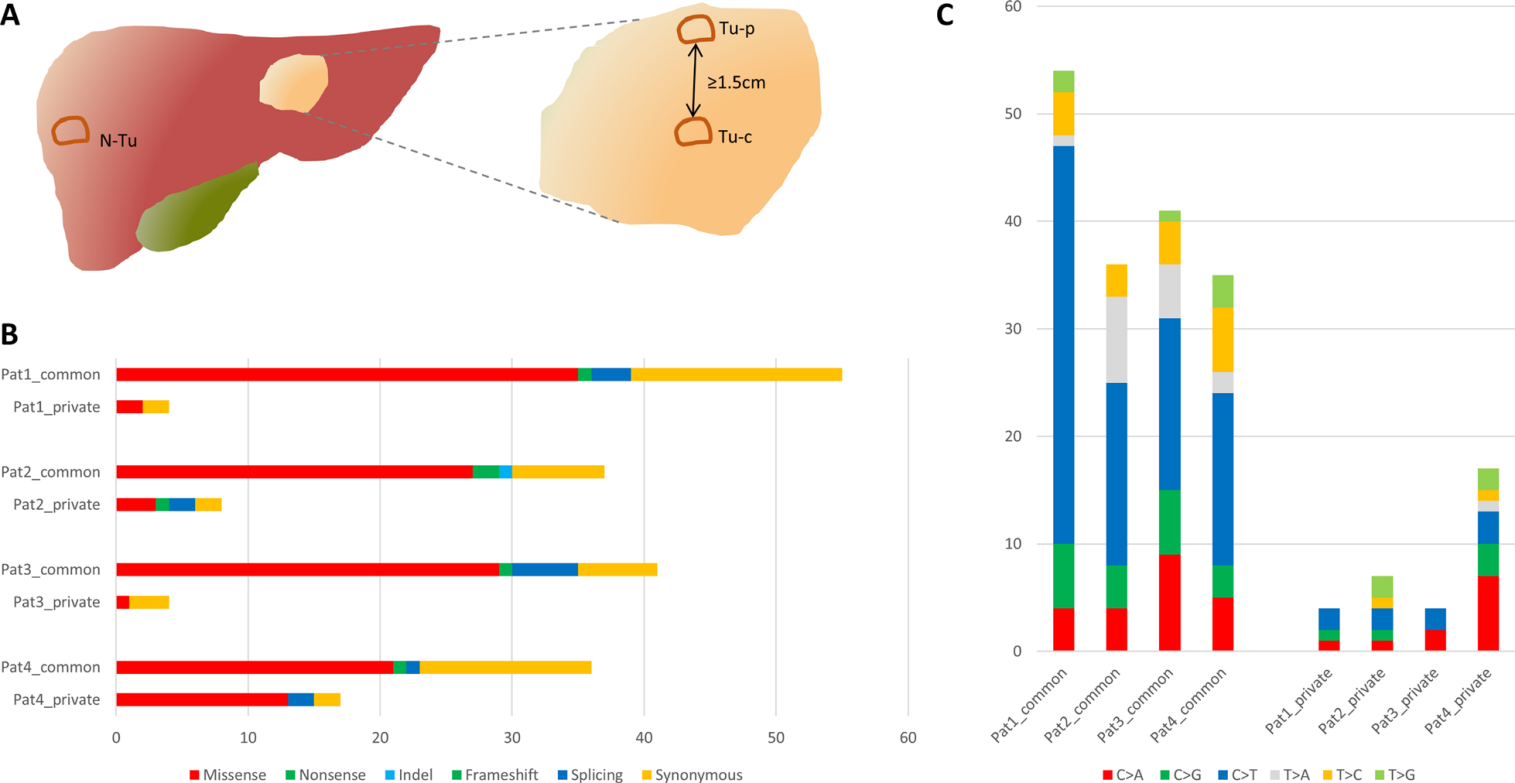
(**A**) Scheme of the tumor regions samples were taken from for whole exome sequencing. (**B**) Mutational spectra of all four patients. (**C**) Substitution pattern of common and private mutations. N-Tu: Non-tumor sample, Tu-p: peripheral tumor sample, Tu-c: central tumor sample.

Exomes of all 12 tumor and non-tumor samples were successfully sequenced. Mean target coverage depth of all patients was 109 (range 87–126). Overall, 208 mutations in 202 genes were identified. Per patient, we detected 38–43 (mean 39.5) non-synonymous and 9–18 (mean 12.8) silent mutations. The most frequent (48%) point mutation was C>T/G>A transition in all patients, whereas 27–55% of these substitutions were found at CpG sites (mean 45%). Functional categories and composition of mutations are shown in Figure 1B, 1C. A case-wise overview of sequencing data is provided in Supplementary Table 1.

### Somatic intratumoral heterogeneity

**Figure 2:**
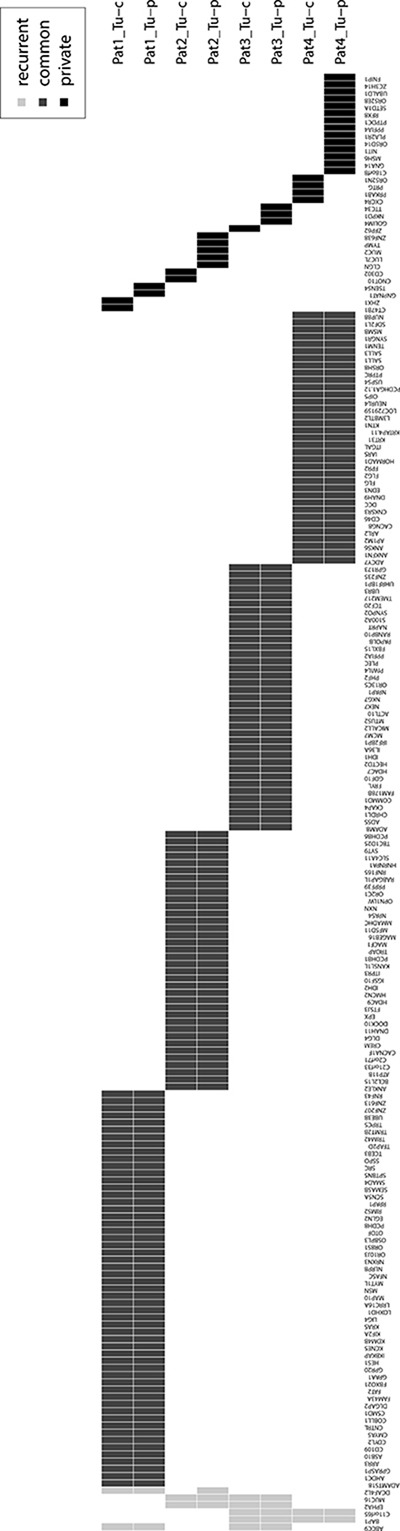
Overview of all recurrent (light grey), common (dark grey) and private (black) synonymous and non-synonymous mutated genes of this study Tu-c: central tumor sample, Tu-p: peripheral tumor sample.

To assess presence of intratumoral subclonality, all mutations were classified into common and private mutations, whereas the latter were unique in one of the tumor regions of any respective patient. These private mutations were found in both samples of patient 1, 2 and 4. In patient 3, private mutations were only identified in the central sample. We identified 0–14 non-synonymous private mutations (mean 3) compared to 24–41 non-synonymous common mutations per sample (mean 33.3), respectively. The mean percentage of private mutations was 12% (range 0–58). Patient 1–3 and the central sample of patient 4 had ≤ 10% private mutations, whereas 58% of private mutations were identified in the peripheral sample of patient 4. Overview of all identified synonymous and non-synonymous mutations is given in Figure 2. In private mutations, no dominating substitution was observed apart from patient 4, where mostly C>A/G>T substitutions were found. The median allele frequency was comparable in common and private mutations (24% vs 22%, Supplementary Figure 1). Of note, a slightly higher median allele frequency was observed in the peripheral sample (range 0.22–0.31) compared to the central one (0.19–0.24) in all patients (Supplementary Figure 2). Representative examples of common and private mutations are shown in Supplementary Figure 3 and all identified mutations are listed in Supplementary Table 2.

### Potential driver mutations and recurrently mutated genes

**Figure 3:**
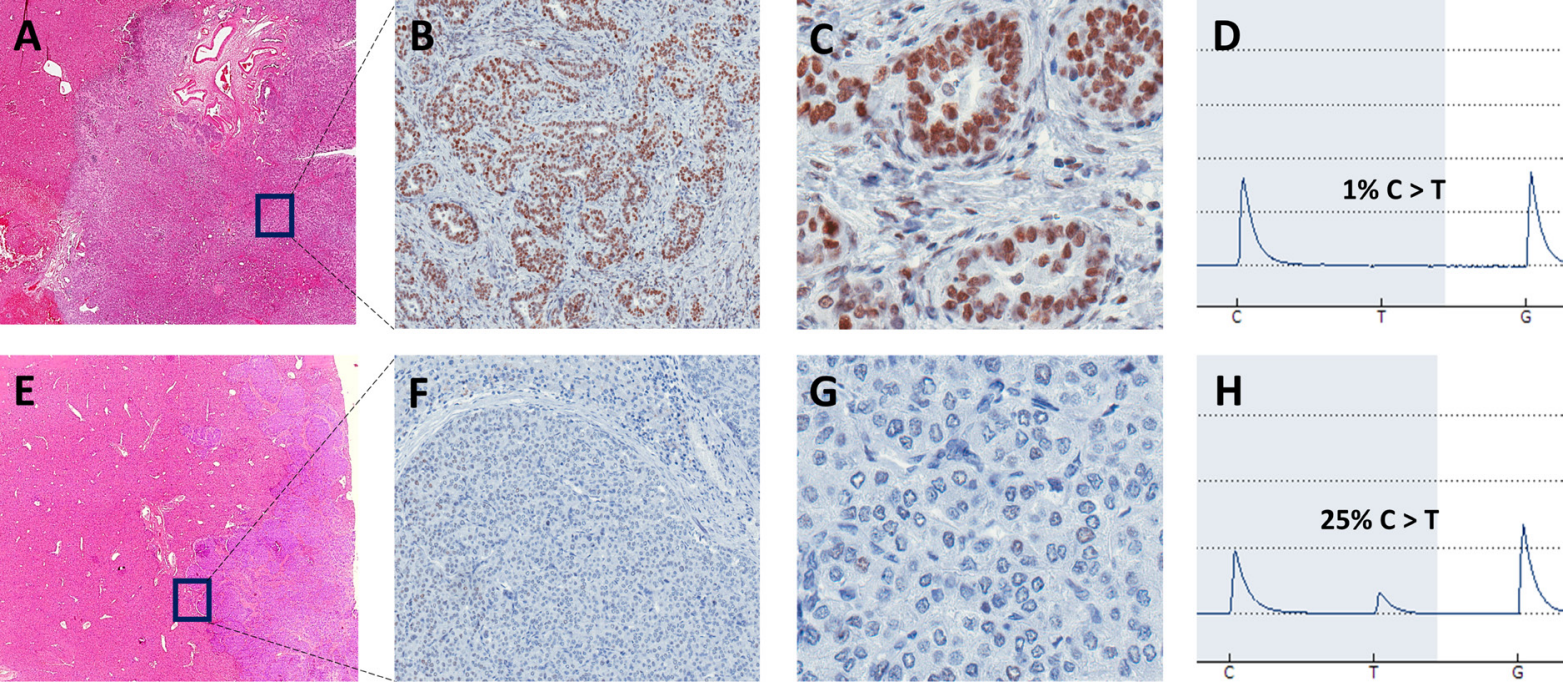
Representative images of HE and MSH6 stained FFPE slides of tumor center (**A**: 60×, **B**: 200×, **C**: 400×) and tumor front (**E**: 60×, **F**: 200×, **G**: 400×) as well as corresponding pyrogram including detected mutation frequency (**D**: tumor center, **H**: tumor front) of macrodissected tissue of patient 4 with C>T missense mutation at position 48010469, chromosome 2. In the tumor center, a glandular growth pattern with clear MSH6 expression and no mutation was observed in contrast to a dedifferentiated growth pattern, no MSH6 expression and 25% of mutant allele frequency at the tumor front.

To analyze a potential difference in tumor growth biology, all tumor samples were investigated for the presence of potential driver mutations (see methods for definition). Twenty potential driver mutations were identified (mean 5, range 4–7): “High confidence driver mutations” were found in patient 1, 2, and 3, while “putative driver mutations” were only found in patient 2, 3, and 4 (Table 1). The only private potential driver mutation was in *MSH6* in the peripheral sample of patient 4. MSH6 is involved in the DNA mismatch repair and mutations of *MSH6* can lead to microsatellite instability (MSI) [14]. Due to the very high amount of private mutations in the peripheral sample of patient 4 compared to all other samples and as the *MSH6* mutation was classified as “putative driver mutation”, we wanted to further study its possible contribution to the pathogenesis and ITH of this tumor. Therefore, immunohistochemistry of fresh frozen tissue as well as FFPE sections obtained by routine diagnostics were performed to study the expression of MSH6. While there was a rather weak staining difference in the fresh frozen material (the MSH6 antibody is recommended for FFPE tissue by the manufacturer), we observed clearly varying expression patterns of MSH6 in peripheral and rather central areas of the tumor in the FFPE sections. After macrodissection of these areas, pyrosequencing clearly confirmed the *MSH6* mutation, identified in the whole exome sequencing, in tumor tissue with lack of MSH6 protein expression. No mutation was found in the area exhibiting MSH6 expression (Figure 3).

**Table 1:** Overview of all common (com) and private potential driver mutations found within this study

Patient	Common / private	Gene	Category	Amino acid change	Relevance	Reason of relevance
**Pat1**	Com	CSMD1	missense	Val2977Met	high	SIFT score < 0.05
**Pat1**	Com	KRAS	missense	Gly12Asp	high	known mutation
**Pat1**	Com	SMAD4	missense	Ser72Thr	high	SIFT score < 0.05
**Pat1**	Com	FAT2	missense	Arg2024Thr	unknown	
**Pat1**	Com	MSN	missense	Val268Ile	unknown	
**Pat1**	Com	MYT1L	missense	Ser696Asn	unknown	
**PAT1**	Com	SRC	frameshift	Thr524HisfsTer52	high	Manual review
**Pat2**	Com	EPHA2	missense	Arg861Cys	high	known mutation
**Pat2**	Com	IDH2	missense	Arg172Trp	high	known mutation
**Pat2**	Com	DNAH11	missense	Glu1074Lys	putative	≤ 5 AA to known mutation
**Pat2**	Com	MUC16	missense	Arg2736Trp	putative	≤ 5 AA to known mutation
**Pat3**	Com	IDH1	missense	Arg132Cys	high	known mutation
**Pat3**	Com	MUC16	missense	Thr2087Met	putative	≤ 5 AA to known mutation
**Pat3**	Com	BAP1	splice site	NA	unknown	
**Pat3**	Com	EPHA2	nonsense	Cys262Ter	unknown	
**Pat4**	Tu-p	MSH6	missense	Arg33Cys	putative	≤ 5 AA to known mutation
**Pat4**	Com	BAP1	frameshift	Asn133GlnfsTer10	putative	≤ 5 AA to known mutation
**Pat4**	Com	DNAH9	missense	Phe4464Ser	unknown	
**Pat4**	Com	GNA14	splice site	NA	unknown	
**Pat4**	Com	PTPRC	missense	Gln407Lys	unknown	

AA: amino acid, Tu-p: tumor peripheral, high: high confidence driver mutations, putative: putative driver mutations, unknown: mutations of unknown relevance. All mutations of unknown relevance were present in the list of genes known to be mutated in biliary tract cancer or present in cancer gene census but did not fulfill criteria for putative or high confidence driver mutations (see methods for details).

Apart from the mutation in *MSH6*, two further mutations in genes known to be mutated in cancer, but rarely in biliary tract carcinoma (*PTPRC, MSN*), were identified as “mutations of unknown relevance”. Moreover, in manual review of non-synonymous mutations, we identified a frameshift mutation in *SRC*, a gene encoding for a tyrosine kinase only rarely described to be mutated in biliary tract cancer and not present in the COSMIC cancer gene census. Usually, this protein is inactivated and becomes activated by dephosphorylation of tyrosine at position 530 [15]. In patient 1, we observed a frameshift at position 524 consecutively leading to an amino acid change at position 530 (tyrosine > valine) and thereby most likely activating SRC (Supplementary Figure 4). Due to this high probability of an oncogenic effect the frameshift mutation in *SRC* was also classified as “high confidence driver mutation”.

Apart from potential driver genes, we investigated a number of recurrently mutated genes in our cohort: three genes known to be frequently mutated in cancer (*EPHA2, BAP1, MUC16*) as well as three genes neither frequently reported in biliary tract cancer nor present in cancer gene census (*DCAF4L2, C11orf65, ABCC9*) were found.

### Copy number variation analysis

**Figure 4:**
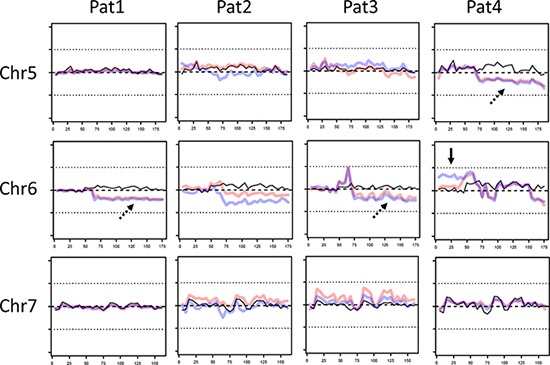
Representative copy number variations (CNV) on chromosomes 5-7 of patient 1-4 Of each patient CNV of non-tumor sample (black line), central tumor sample (red line) and peripheral sample (blue line) is shown. In some fractions of the genome CNV between both tumor samples and control were observed (dotted arrow), whereas in chromosome 6 the copy numbers between both tumor samples varied. X-axis: chromosome length (megabases), y-axis: deviation from expected coverage, upper dotted line = +50%, lower dotted line = −50%.

To analyze ITH of copy number aberrations, copy numbers of both tumors and the corresponding normal sample were calculated and compared to a set of reference samples to reduce bias introduced by the target enrichment method. A fraction of the exome showed copy number changes in all tumors in comparison to the normal sample. For instance, we observed amplified regions in chromosome 1p36.3-1p11 (patient 1, 3 and 4) as well as in chromosome 16p13.3-16p11.1 of patient 1. A heterozygous deletion was observed in chromosome 6q11-6q27 in patient 1 and 3, respectively. Furthermore, copy numbers varied between different tumors. However, the only clear sign of ITH was found in chromosome 6p of patient 4, where our data suggest a heterozygous duplication in the area of mega base 0–50. Of note, in this area on chromosome 6p21.1, the signal protein vascular endothelial growth factor (VEGF) is located, which stimulates angiogenesis and is therefore able to contribute to tumor growth when overexpressed. No other potentially oncogenic genes were identified in this duplicated region. Representative data of chromosome 5–7 is shown in Figure 4 (full copy number profiles are provided in Supplementary Figure 5, raw data is available on request).

## DISCUSSION

Reasons for the lack of successful targeted therapy studies in iCCA are intensively debated. Data on ITH as possible bias for biopsy based therapy approaches is missing to date. In the present study we investigated mutational profiles based on whole exome sequencing of four patients and thereby discovered a considerable degree of ITH in iCCA and could demonstrate influence of a private mutation in *MSH6* on spatial mutational burden.

We identified a mean of 38 non-synonymous mutations per patient, which is comparable to other large scale sequencing studies on CCA [16–18]. Likewise, we predominantly observed C>T/G>A transitions as somatic substitution patterns, which was reported in various cancer types [19]. The main aim of this study was to investigate presence and extent of ITH in iCCA. We identified private mutations in all four patients, while the mean share of private mutations was 12% (range 0–57%) of non-synonymous mutations per sample and 1/20 (5%) of potential driver mutations was found to be private. These findings indicate a substantial amount of ITH in intrahepatic cholangiocarcinoma. The amount of heterogeneity seems to be lower than in lung cancer, where 76% of mutations were reported to be common in a large scale sequencing study and far lower than clear cell renal cancer, where 73–75% of driver aberrations were subclonal [11, 13]. Likewise, in prostate cancer and primary breast cancer, a higher amount of ITH was reported [20, 21]. The high amount of driver mutations found to be common is comparable with data on lung and breast cancer, where the majority of driver mutations was common [11, 21]. However, when comparing studies exploring ITH, it has to be considered that the degree of ITH was reported to increase with the number of biopsies examined and the low number of samples in this study may underestimate the true rate of ITH in iCCA [13]. Notably, we observed a slightly higher allele frequency in the peripheral compared to central samples (Supplementary Figure 2). This is most likely due to a higher tumor content in the peripheral sample deriving from a stronger degree of desmoplasia in the tumor center. This important feature of iCCA has to be considered in data interpretation since it might have hindered detection of variants with low allele frequency in the central samples.

As reported in other whole exome studies on CCA we observed several copy number changes comparing tumor and non-tumor copy numbers based on sequencing coverage depth [16–18]. Notably, the only sign of ITH was chromosome 6p in patient 4, where we observed a heterozygous duplication. An enhanced expression of *VEGF*, which is located on 6p, may promote to tumor growth, but does not explain the high amount of private mutations in patient 4. The low amount of ITH in copy numbers observed in the current investigation is in line with findings on lung cancer, but unlike clear cell renal carcinoma, where a substantial amount of ITH in copy numbers could be demonstrated [11, 13]. One has to bear in mind when interpreting this data, that calculation of copy numbers based on whole exome data is limited due to missing information of non-coding regions.

The only sample in this study with a markedly higher amount of private mutations compared to all other samples (57% vs ≤ 10%) was the peripheral sample of patient 4. Of note, a missense mutation in the DNA mismatch repair protein MSH6 could be detected in this sample and varying expression patterns of MSH6 could be shown in immunohistochemistry. Furthermore, in contrast to C>T/G>A dominated common mutations, in patient 4, G>T/C>A substitution was the most frequent SNV. This is remarkable since the MSH2-MSH6 heterodimer is known to have a high affinity to G/T-mismatches [19, 22, 23]. We therefore conclude that the private mutation in MSH6 may have led to spatial MSI and a consecutively increased mutation rate, which is in line with a study on prostate cancer, where somatic mutations in MSH6 have been associated with hypermutation [24].

Of note, we observed no marked difference in the indel frequency between the peripheral sample of patient 4 and the other samples and mutation count of this sample was not as high as described for hypermutated tumors [24, 25]. This could be explained due to a short period between the hit of MSH6 mutation and surgery: the MSH6 mutation might have been a late event in the oncological development. Another possible explanation for the indel frequency might be that MSH6 was the only MMR protein found to be mutated. Data on prevalence of heterogenic MSH6 expression in iCCA are lacking to date, but it was described to be present in other neoplasms such as colorectal and endometrial carcinomas [26, 27]. The clinical relevance of *MSH6* heterogeneity in iCCA is underlined by studies reporting resistance to alkylating agents in gliomas due to *MSH6* mutation [28, 29]. This is of high interest, since current standard therapy of iCCA includes platinum, an alkylating-like acting agent, and MSI was reported to be present in 16–49% of iCCA [3, 30, 31]. Moreover, recent data reported remarkable clinical responses to anti-PD1 immune check-point treatment of cancers with MSI [32, 33]. These findings highlight the consequences on clinical management derived by ITH and warrant further investigation of MSH6 heterogeneity in CCA. Furthermore, ITH of potential driver mutations should be considered in the design of future personalized therapy trials based on mutational profiles of single biopsies. Likewise, when establishing subclasses of iCCA based on mutational and gene expression data from single biopsies, ITH should be taken into account. Despite that, further studies investigating more samples at more time points are warranted to better characterize spatial and temporal ITH in iCCA.

Besides the mutation in *MSH6*, we identified 3 more mutations affecting genes described in cancer, but only rarely reported in biliary tract cancer (≤ 2%; *SRC, MSN, PTPRC*). In the non-receptor tyrosine kinase SRC, a frameshift mutation involving a regulating phosphorylation site at the end amino acid sequence was observed. Since activating frameshift mutations in regulating domains have already been described such as for NOTCH, we hypothesize that this mutation is likely damaging [34–36]. SRC plays a major role in different aspects of oncogenesis and was described to be mutated in various human cancers [37–40]. Moreover, a SRC family tyrosine kinase inhibitor (Dasatinib) was already approved for treatment of chronic myeloid leukemia stressing the need of further studies on prevalence of activating *SRC* mutations in iCCA [41].

Taken together, our study indicates that iCCA has a substantial amount of ITH, which is important to consider in planning targeted therapy trials based on single biopsy mutation profiles. Private mutations in DNA mismatch proteins like MSH6 most likely lead to a significant gain of mutational burden in circumscribed regions of the tumor and thereby might have crucial impact on clinical management.

## MATERIALS AND METHODS

### Patients and sample preparation

Surgical specimens of four patients with resection of iCCA in curative intention were obtained between 11/2015 and 01/2016. Mean age was 71 (range 67–86), 3/4 patients were female and all patients were Caucasians. iCCA were staged according to the 7th edition of the classification of the Union for International Cancer Control (UICC). 3/4 patients had an advanced tumor stage with lymph node metastasis (stage IVA), whereas one patient was staged as UICC I. Mean follow up was 6.8 months (range 6–8 months), and one patient had recurrent disease in follow up (patient 2). Biomaterial and clinical data were obtained from biobank and the tumor documentation of the UCT Frankfurt (University Cancer Center, Frankfurt, Germany). Informed consent was obtained from all patients before surgery. The study protocol was approved by the local ethics committee of the University of Frankfurt (Approval No. SGI-05-2016). Clinicopathological characteristics are provided in Table 2.

**Table 2:** Clinicopathological characteristics including TNM classification and staging according UICC (Union for International Cancer Control), 7th edition

Case	Age	Sex	Diameter (cm)	Distance (cm)	T	N	M	L	V	pN	G	R	UICC
**Pat1**	69	F	8.5	4	1	×	0	0	0	0	2	1	IVA
**Pat2**	67	F	10	5	2b	1	0	0	0	1	2	1	IVA
**Pat3**	86	F	4.7	1.7	3	×	0	0	0	0	3	0	IVA
**Pat4**	60	M	5	1.7	1	0	0	0	0	0	2	0	I

All tumors were inspected at initial assessment in the pathology department and tissue was stored in liquid nitrogen if a peripheral and central sample with at least 1.5 cm distance could be obtained (Figure 1A). Mean tumor diameter was 7.1 cm (range 5–10 cm) and mean distance between central and peripheral sample was 3.1 cm (range 1.7–5 cm). A matched normal sample from healthy liver tissue was obtained as well. Only tumors with definite diagnosis of an iCCA, confirmed by two expert gastrointestinal pathologists, were included. Patient 1 and 2 had a predominantly glandular growth pattern, whereas patient 3 and 4 had a partly solid growth pattern as well. Percentage of tumor content was assessed based on hematoxylin and eosin stained frozen sections by an expert gastrointestinal pathologist. In cases of clearly varying tumor content within one section, laser capture microdissection (LCM) was performed to enhance the percentage of malignant tissue (*n* = 4). For LCM, areas with comparably high tumor content were isolated from 4–6 μm tissue slides with the PALM MicroBeam IV Laser Capture System (ZEISS, Oberkochen, Germany). Final estimated mean percentage of tumor cells was 47.5% (range 35–70%). Data on tumor content and representative histological sections are shown in Supplementary Figure 6. DNA was extracted with QIAamp DNA Micro Kit (Qiagen, Hilden, Germany) from fresh frozen material and laser dissected samples according to manufacturer's recommendations. DNA yield was quantified with Quantus Fluorometer (Promega, Madison, WI).

### Whole exome sequencing

Sequencing libraries were prepared from tumor and non-tumor tissue with SureSelectXT Human All Exon V6 (target size 60 Mb, Agilent, Santa Clara, CA) according to the manufacturer's instructions and paired-end sequencing was performed on a HiSeq2500 (Illumina, San Diego, CA) with 2 × 100 base pairs (bp) read length. At least 10 gigabases of raw read data per non-tumor sample (*n* = 4) and 14 gigabases per tumor sample (*n* = 8) were produced.

### Variant calling

Illumina CASAVA (1.8.2) was used for demultiplexing of sequenced reads and Skewer (Version 0.1.116) for adapter trimming. All following steps of raw reads analysis were performed with Genomatix Mining Station (GMS), Genomatix Genome Analyzer (GGA) and Genomatix GeneGrid (Genomatix Software GmbH, Munich, Germany). The trimmed reads were aligned with GMS (Version 2.4.1, Genomatix Mapper Version 3.7.6.3) onto human genome reference sequence (NCBI build 37) with mapping type deep and mapped with insertion and deletion option (minimum quality ≥ 92%).

For detection of SNVs and small insertions and deletions (indels) SAMtools was used [42]. First SAMtools mpileup was used to compute the likelihood of a variant given the observed data and specific quality parameters. The Small Variant Detection program then applied bcftools for variant calling and SAMtools script vcfutils.pl was used to filter the data. To enhance sensitivity, variant calling was performed with two mutation calling algorithms: Genomatix GeneGrid and VarScan 2.4.2 were used for extensive annotation and interpretation of all SNVs and small indels.

The following cut-offs were used for detection of somatic single nucleotide variants (SNV): (1) mapping quality ≥ 20, (2) variant allele frequency in the tumor ≥ 5%, (3) general sequence depth ≥ 10 both non-tumor and tumor samples, (4) tumor variant sequence depth ≥ 2, (5) non-tumor variant sequence depth ≤ 1, (6) no potential indel within 5 bp of the suspected SNV, (7) not more than 2 SNVs in any 10-bp window. Additionally, all mutations were confirmed by inspection of the sequencing data in the genome browser and all SNVs with suspicion for sequencing or alignment error were removed. Indels were analyzed differently and only high quality indels were further investigated: (1) minimum coverage of 20 in both tumor and non-tumor sample, (2) minimum allele frequency of 10% and (3) a minimum of at least 5 independent reads of the mutation as well as (4) no read in the non-tumor sample.

Variants were compared to the 1000 genomes project data base and common polymorphisms were excluded. Only SNVs in exons or splice sites were further analyzed. To investigate presence of subclonality, somatic SNVs of both central and peripheral tumor samples were compared. For each mutation found only in one tumor sample, sequencing data of the corresponding tumor sample was investigated for presence of reads with the same information. All SNVs with suspicion for private mutations were subjected to validation with pyrosequencing. Mutations that could be validated to be present only in the tumor center or periphery were regarded as private, whereas mutations present in both samples were regarded as common.

### Sanger sequencing

Detected somatic mutations found in both tumor samples were validated with Sanger sequencing. Potential driver mutations (definition see below), doubtful SNVs and randomly selected highly probable true SNVs were subjected to Sanger (for validation algorithm, see Supplementary Figure 7). Primers were designed using NCBI Primer-Blast (http://www.ncbi.nlm.nih.gov/tools/primer-blast). PCR reaction was performed with *Taq* PCR Master Mix Kit (Qiagen, Hilden, Germany) according to manufacturer's recommendations using 20 pmol primer and 25–50 ng template DNA. PCR reaction conditions were initial denaturation at 95°C for 300s, 44 cycles of 95°C for 45s, 56–61°C for 60s and 72°C e for 45s, followed by 5 min final extension at 72°C. The annealing temperature was chosen suitable for the respective primer pairs. PCR amplification was always performed for central and peripheral tumor as well as the non-tumor sample. PCR solutions were sent to Eurofins Genomics GmbH (Ebersberg, Germany) for sequencing. Primer sequences are listed in Supplementary Tables 3 and 4.

### Pyrosequencing

For all SNVs suspicious for private mutations in manual inspection in the genome browser, pyrosequencing of both tumor samples and corresponding non-tumor sample was performed. Primer design was performed with PSQ Assay design (Biotage, Uppsala, Sweden) and assays were designed with Pyromark Q24 (Qiagen, Hilden, Germany). PCR reaction was performed with the PyroMark PCR Kit (Qiagen, Hilden, Germany) according to manufacturer's recommendations using 20 pmol primer and 25–50 ng template DNA. The PCRs were performed as described for Sanger sequencing. The resulting PCR products were sequenced with the PyroMark Q24 pyrosequencer using PyroMark Gold Q96 reagents (Qiagen, Hilden, Germany). All assays were run with tumor and non-tumor samples as well as positive (Qiagen human control DNA) and negative control. SNVs with ≥ 5% difference in mutant allele frequency compared to non-tumor tissue and positive control were assessed as true variants. Pyrosequencing results for private mutations are provided in Supplementary Table 5.

50/51 (98%) SNVs selected for validation with suspicion for a common mutation and 25/41 (61%) SNVs with suspicion for a private mutation were successfully validated. We initially re-sequenced several SNVs with a rather weak suspicion for private SNVs which retrospectively appear as artefacts. These SNVs are included in the validation rate for private mutations as well potentially causing the low rate of successful validation in the group of mutations. Six Mutations were excluded since no specific primers could be established.

### Identification of potential driver genes

A selection of recurrently mutated genes in large scale or targeted sequencing projects on iCCA (all genes mutated at least twice were included [17, 18, 43–49]) as well as genes already described to be mutated in ≥ 3% of biliary tract cancer in the COSMIC database (data extracted May 2016) or present in the COSMIC cancer gene census (data extracted July 2016) was created. This selection was matched with all genes with non-synonymous mutations in this study. Mutations present in the resulting list were categorized as possible drivers for iCCA and examined in more detail whether the amino acid substitution has already been described: “High confidence driver mutations” were defined as mutations already described in COSMIC database or having a SIFT score of < 0.05, whereas mutations leading to amino acid changes in close proximity (≤ 5 bp) to known mutations were classified as “putative driver mutations” [50]. All other mutations were classified as “mutations of unknown relevance”. Besides matching all non-synonymous mutations with the established list, all mutated genes were manually reviewed and those with high probability to have an oncogenic effect were examined in more detail.

### Copy number analysis

Copy number variations (CNV) were computed on uniquely mapping, non-duplicate, hiqh quality reads using an internally-developed method based on sequencing coverage depth. Briefly, we used at least 10 reference samples to create a model of the expected coverage that represents biases introduced by the target enrichment method, sequence GC content, library preparation protocol, insert size and sequencing technology, as well as inter-sample variation.

CNV calling for germline samples was performed by computing the sample's coverage profile, correcting for total read count and computing the deviation from the expected coverage. Genomic regions were called as variant if they deviate by at least 2 standard deviations from the model mean and the deviation was concordant with a biologically possible copy number (e.g., +50% for a heterozygous duplication, −50% for a heterozygous deletion). For tumor samples, the estimated tumor content had to be taken into account to deduct the copy number. For instance, given a tumor content of 60%, an observed deviation of +30% represented a heterozygous duplication in the tumor, while an observed deviation of +20% could either represent a heterozygous duplication of non-tumor tissue or a subclonal duplication in the tumor. The coverage deviation data (not adjusted for tumor content) of all three samples of one patient was combined in a single plot. To improve visual clarity and highlight large-scale changes, data was smoothed using the median over windows of 5 mega bases. Copy numbers were additionally calculated with Genomatix Genome buchstabendreher, resulting in the same pattern of copy number alterations.

### Immunohistochemistry

Formalin-fixed, paraffin-embedded tissue blocks were cut into 2 μm sections and transferred to glass slides. After drying overnight at 37°C, slides were deparaffinized with xylene, rehydrated with ethanol, washed and subjected to a water bath for antigen retrieval for 30 min at 94°C, pH 8 (Trilogy-solution 1:100; Cell Marque Corporation, Rocklin, USA). The slides were then processed on the Autostainer Link 48 (Dako, Glostrup, Denmark) using an automated staining protocol (Dako EnVision™ Flex, K8000). Staining with primary antibody MSH6 (clone SP93, 1:100, DCS, Hamburg, Germany) was performed for 30 min according to manufacturer's recommendations. Counterstaining was performed with hematoxylin.

### Statistics

Descriptive statistics were calculated using BiAS (version 11.01, BiAS for Windows; Epsilon-Verlag, Frankfurt, Germany).

## SUPPLEMENTARY MATERIALS FIGURES AND TABLES




