# Supplementary material for: Intratumoral heterogeneity of intrahepatic cholangiocarcinoma
Source: Oncotarget. 2017 Jan 27;8(9):14957–68. doi: 10.18632/oncotarget.14844 (PMC5362457; doi:10.18632/oncotarget.14844)
Supplement: Supplementary file 1 [file oncotarget-08-14957-s001.pdf]

Intratumoral heterogeneity of intrahepatic cholangiocarcinoma

Supplementary Materials

Supplementary Table 1: Mean coverage and percentage of bases covered at least 10x and 20x

Patient	Sample	Mean Coverage	10X	20X
**Pat1**	N-Tu	90.3	94.5	90.7
**Pat1**	Tu-c	125.5	95.7	93.5
**Pat1**	Tu-p	125.3	95.5	93.1
**Pat2**	N-Tu	87.4	94.4	90.4
**Pat2**	Tu-c	122.5	95.5	93.2
**Pat2**	Tu-p	98.4	91.8	85.9
**Pat3**	N-Tu	100.6	94.7	91.2
**Pat3**	Tu-c	98.4	91.8	85.9
**Pat3**	Tu-p	122.5	95.5	93.2
**Pat4**	N-Tu	102.6	95	91.7
**Pat4**	Tu-c	113	95.6	93
**Pat4**	Tu-p	125	95.7	93.3
**Mean**		119	95	93

Supplementary Table 2: All detected common (com) and private mutations in either central (Tu-c) or peripheral (Tu-p) tumor sample. see Supplementary_Table_2

Supplementary Table 3: Primer sequences for all genes validated with Sanger sequencing

Patient	Gene	Forward Primer (5′ > 3′)	Reverse Primer (5′ > 3′)
**Pat1**	OTOF	CAAGAGGCTTCTGGGTTGTCT	GTTGCCACGGTAGATCTGGT
**Pat1**	ZNF207	AACTTCATTTCAGCCACAGCC	ATCATTACTAGGCACAAGCCTACAG
**Pat1**	SCN5A	TGTCTGAGCCACTCCGTATC	AAAGAGCGTTGCAGCAGGTG
**Pat1**	DCAF4L2	ACCCGTGCATGTGAACGAA	GGATGGGATCTACAAACACCGA
**Pat1**	CSMD1	TTGCCATTTTCATGCTGGGTC	TAACTTGTGCAGTCGGGAGC
**Pat1**	SRC	GTTAGGCTCTCTCGATGGTCC	TCTCAGGCCACCCTAGGTC
**Pat1**	KRAS	AGCGTCGATGGAGGAGTTTGTA	CCCTGACATACTCCCAAGGAAAG
**Pat2**	ITPR3	ACGAGTACCTGAGCATCGAGT	CACCCAGTGCTATTCGGGG
**Pat2**	KANSL1L	GTTGACTGTTTCCTTGCAGAGTG	CGACAGCATGGAGCTCAAGAAT
**Pat2**	ZNF638	TTGTAGCATCCGCTTCAGTCA	TCTCTGGGCACAGATTTTGGAA
**Pat2**	DNAH11	CCTGGAGACCCACACTTACCT	TTCATGTCCACCTTGAACCAACT
**Pat3**	EPHA2	CACCCGCAAAGGCTTCTACC	ACTAATGTGTGTCAAAGCAGGGA
**Pat3**	IDH1	TGATGCCACCAACGACCAAG	GTGTTGAGATGGACGCCTATTTG
**Pat3**	MUC16	CCTGGCTATGGGACCTCACTA	AAAGACCTGTGACCGAGGATG
**Pat3**	TMEM217	AGCTAGTCACTTGGACCCTGT	ATTCCCAGACGGACAGGTAGT
**Pat3**	GDF10	CAGAGATACGACCCCTTCCCT	CAGCCGATGTCTGCGAAGT
**Pat3**	NEK7	TGTGATTATTTTCTACCCCCGTA	GGCCAATTTTACACCACATGCT
**Pat3**	UBR3	CCACACCTACTACAGTTGCCT	AGATGGCTTGACCATATTTTGTG
**Pat3**	CHRDL1	TATGATCCTCCACCAAGCCG	AGGGACCGGACTATGCTCTT
**Pat4**	ATM|C11orf65	ACAGCTTGTTAAGGTGAGCCT	ACGAGACCCGGTATACTGAT
**Pat4**	BAP1	AGGCCATGTTGCTTCCTAGTG	AGCTGACAAAGTGGAACGCC
**Pat4**	SALL1	AAGACAGAGGTCAACGGCT	GATAATCAATGGCAGTGGGACAG
**Pat4**	AP1M2	CTGGACTGTGGGTGTAAGGAAC	GAAGAGCACGCGGTCATTG

Supplementary Table 4: Primer sequences of genes validated with pyrosequencing. Asterisk is indicating the biotinylated primer. see Supplementary_Table_S4

Supplementary Table 5: Overview of variant allele frequencies (VAF) of private mutations as well as corresponding tumor (C-Tu) and non-tumor or positive control (N-Tu) tissue detected with pyrosequencing. Tu-p: peripheral tumor sample, Tu-c: central tumor sample

Patient	Location	Gene	VAF private (%)	VAF C-Tu (%)	VAF N-Tu (%)
**Pat1**	Tu-p	GNPNAT1	15	5	7
**Pat1**	Tu-c	CT47B1	16	4	2
**Pat2**	Tu-p	LUC7L	8	3	3
**Pat2**	Tu-p	MUC2	12	0	0
**Pat2**	Tu-c	DCAF4L2	19	3	1
**Pat2**	Tu-c	CNOT10	31	7	6
**Pat2**	Tu-c	CD302	17	3	5
**Pat2**	Tu-p	TYMP	32	3	1
**Pat3**	Tu-p	GOLIM4	24	2	0
**Pat4**	Tu-c	PRKAB1	12	0	0
**Pat4**	Tu-c	PRTG	8	2	2
**Pat4**	Tu-p	MSH6	29	6	7
**Pat4**	Tu-p	OR5D14	28	1	0
**Pat4**	Tu-p	PLA2R1	22	4	2
**Pat4**	Tu-p	RFX8	25	3	2
**Pat4**	Tu-p	SETD1A	24	3	2
**Pat4**	Tu-p	C18orf8	37	1	1
**Pat4**	Tu-p	GNA14	23	1	1
**Pat4**	Tu-p	NIT1	22	1	1
**Pat4**	Tu-p	PPFIA4	21	1	1
**Pat4**	Tu-p	PTPDC1	28	0	0
**Pat4**	Tu-p	OR52E8	56	24	25
**Pat4**	Tu-p	UBALD1	35	7	7
**Pat4**	Tu-p	ZC3H14	36	5	3
**Pat4**	Tu-p	FNIP1	31	7	4

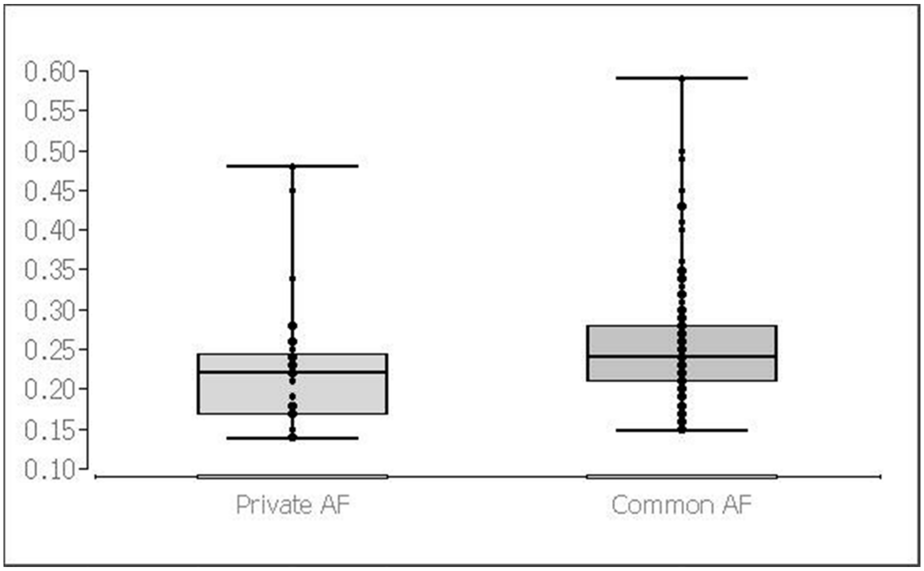

Supplementary Figure 1: Allele frequencies (AF) of private (left) and common mutations (right). For common mutations mean of central and peripheral sample was calculated. Data is shown as median and Q1, Q3. Single values are resembled by dots. Supplementary Figure 3: Validation examples of private and common mutations.

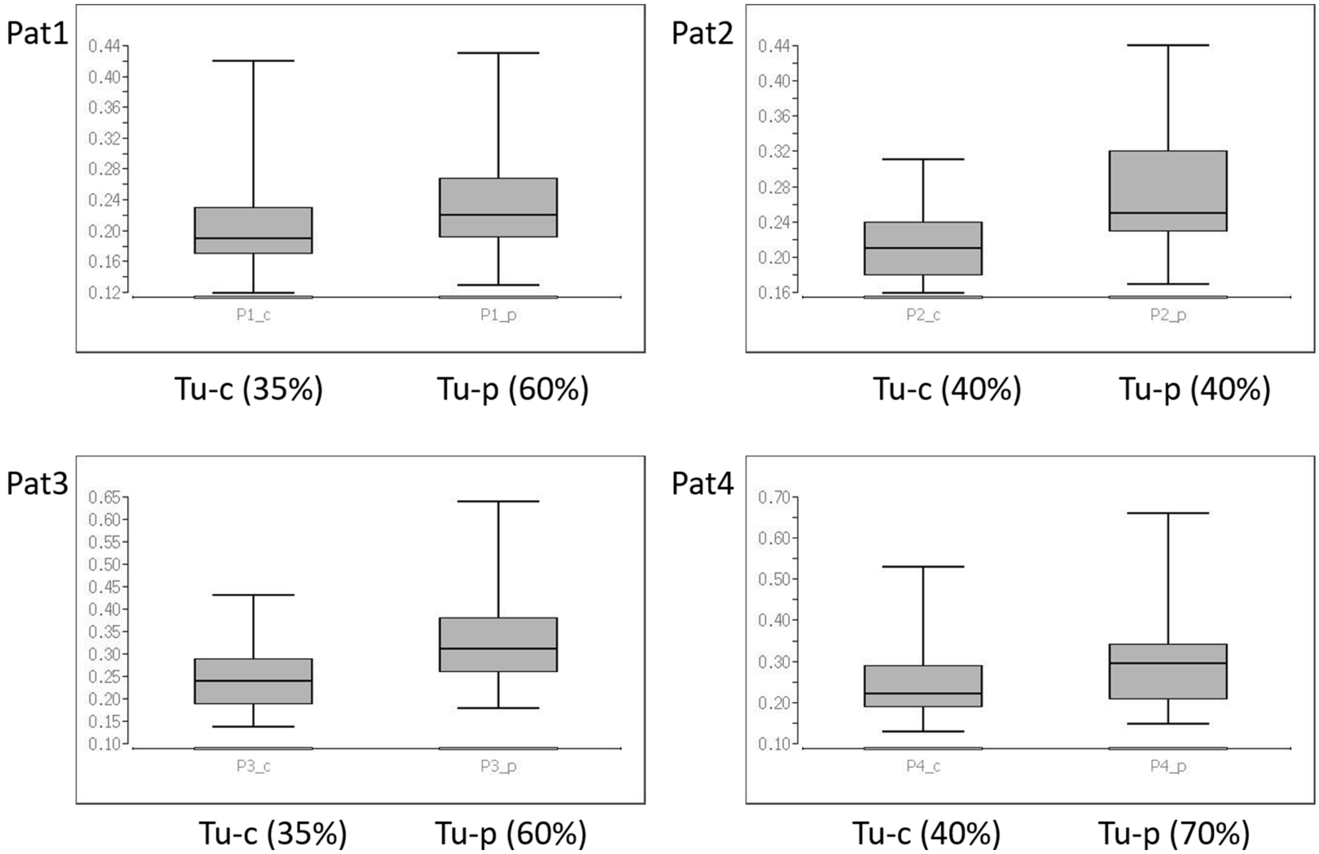

Supplementary Figure 2: Allele frequencies (AF) of mutations in the central (Tu-c) and peripheral (Tu-p) tumor sample. For this analysis all mutations were included with available allele frequency of central and peripheral sample. The black line in the middle of the grey box (Q1-Q3) represents the median. For each sample, estimated tumor content is denoted in brackets.

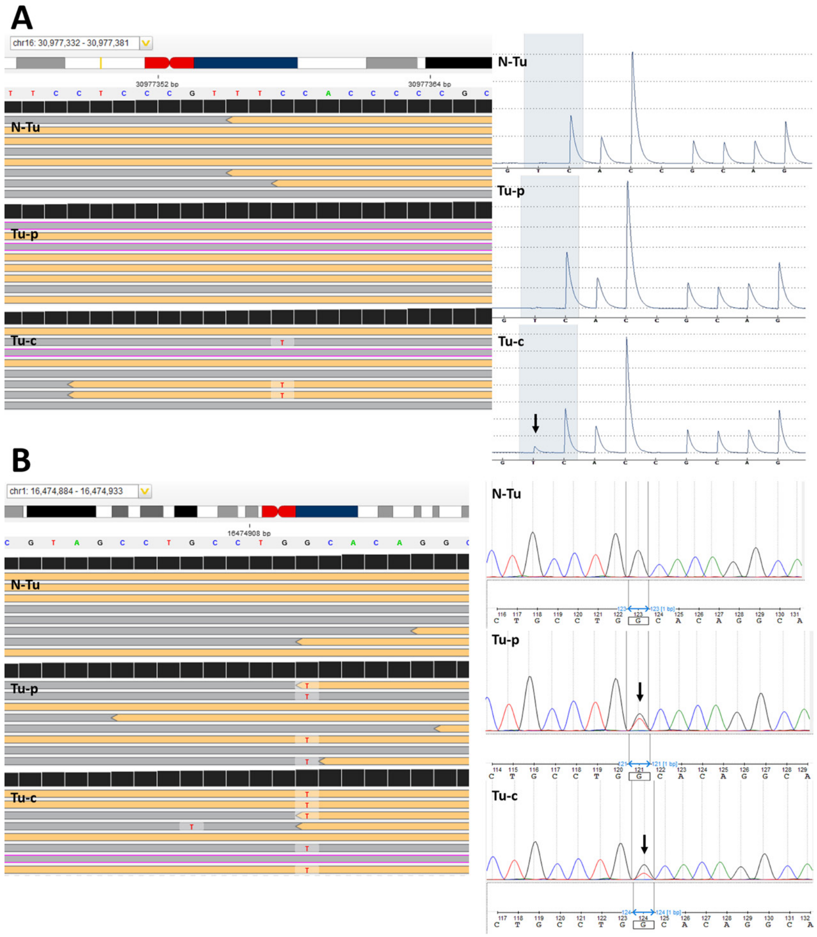

Supplementary Figure 3: (**A**) Example of Private Mutation: Pat4_SETD1A; 16: 30977357, C>T: Missense mutation in Tu-c, (**B**) Example of common mutation: Pat3_EPHA3: 1:16474910, G>T: Nonsense Mutation in Tu-c and Tu-p. Arrows indicate point mutation in pyro (A) and Sanger sequencing (B), respectively.

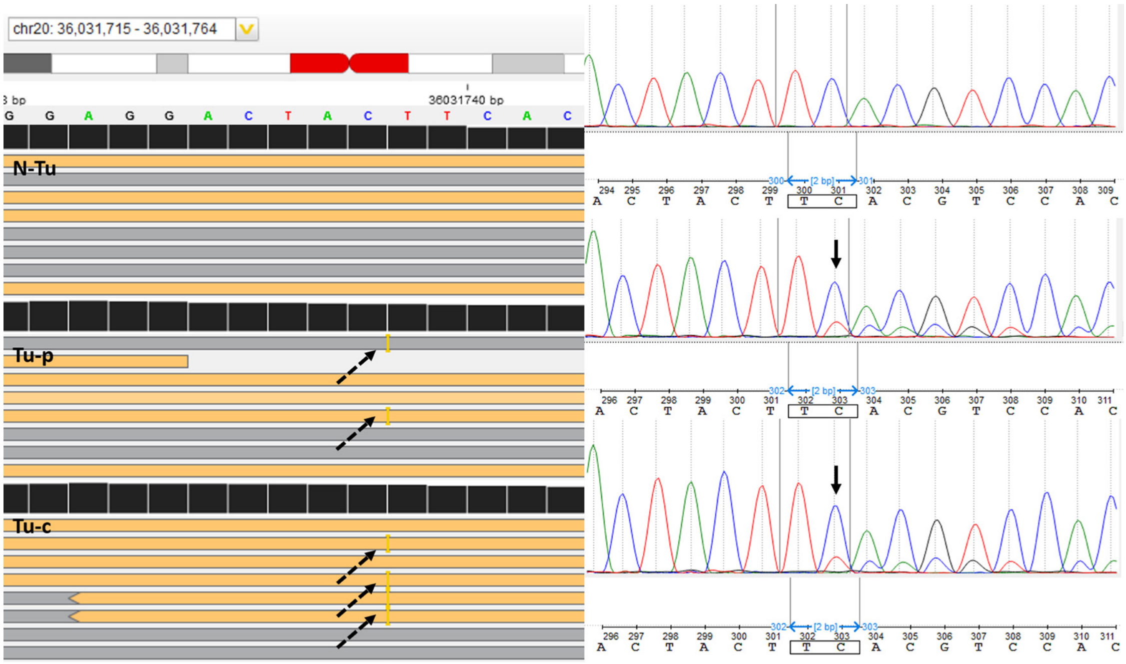

Supplementary Figure 4: Frameshift mutation in Pat1_SRC: 20: 36031737, CTT > CTTT, Thr524HisfsTer52. Dotted arrows indicate the insertion in genome browser (left), arrows indicate start of the frameshift in Sanger sequencing (right).Supplementary Figure 5: Copy number profiles of all patients.

Pat1 Coverage deviation

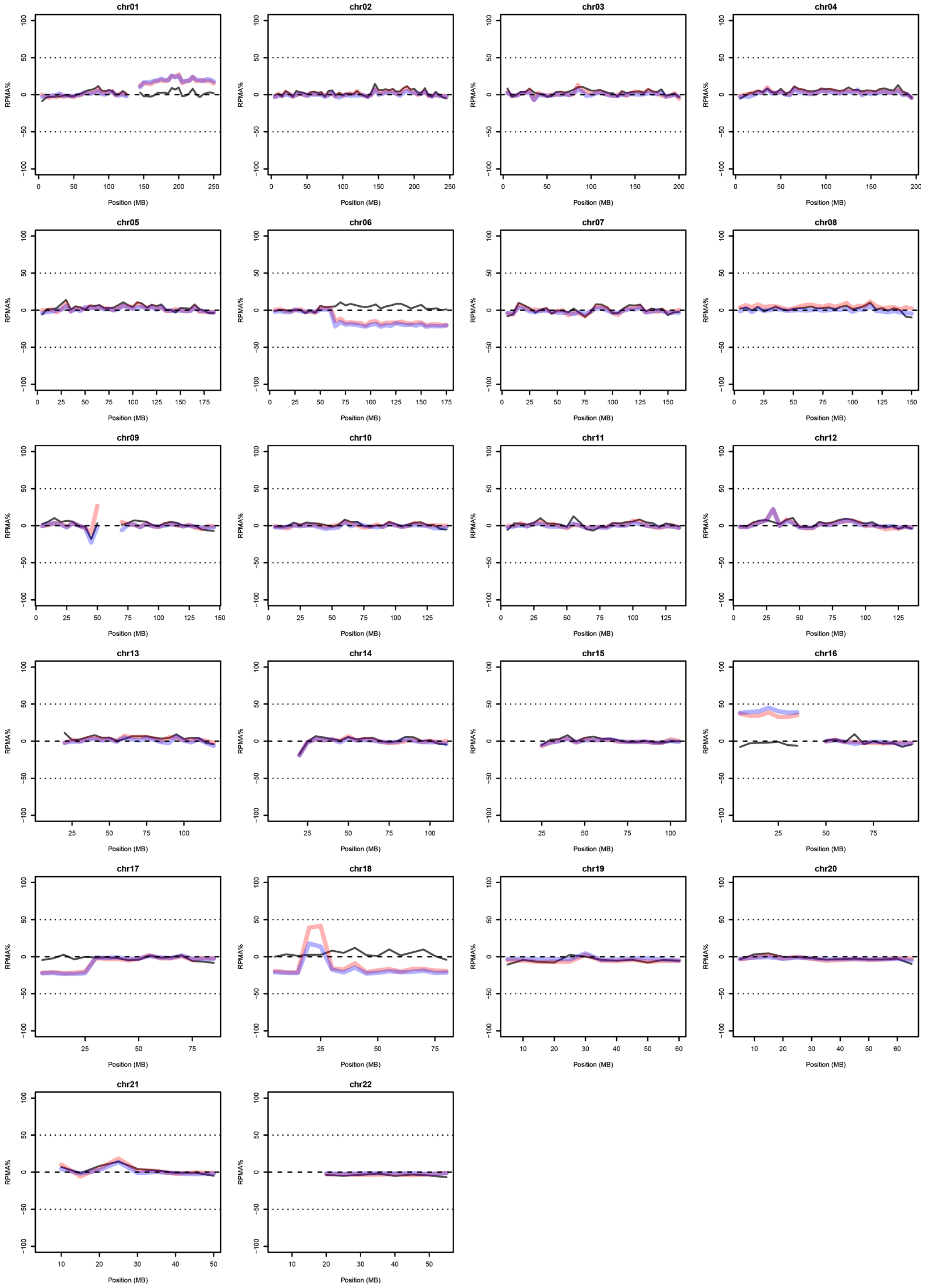

Pat2 Coverage deviation

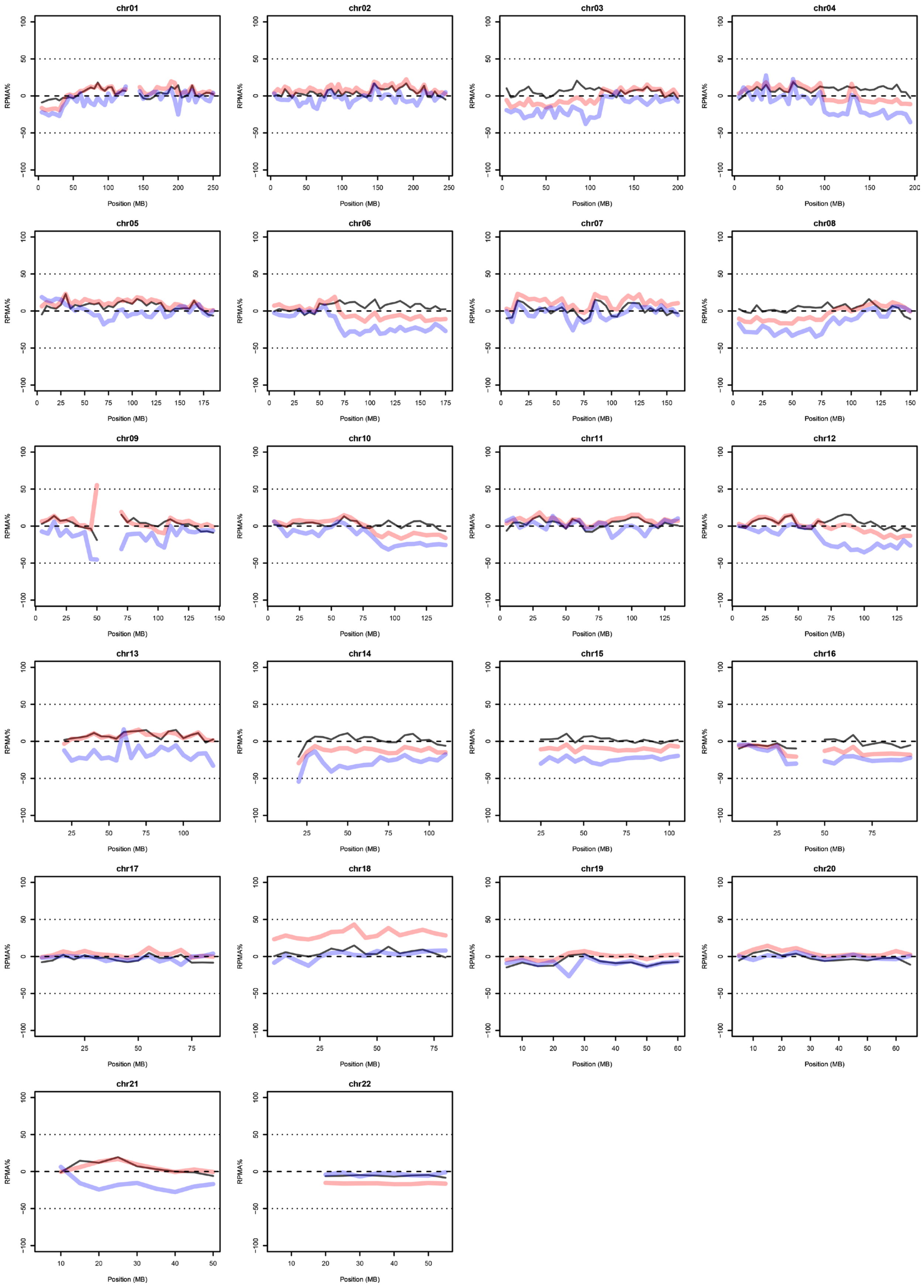

Pat3 Coverage deviation

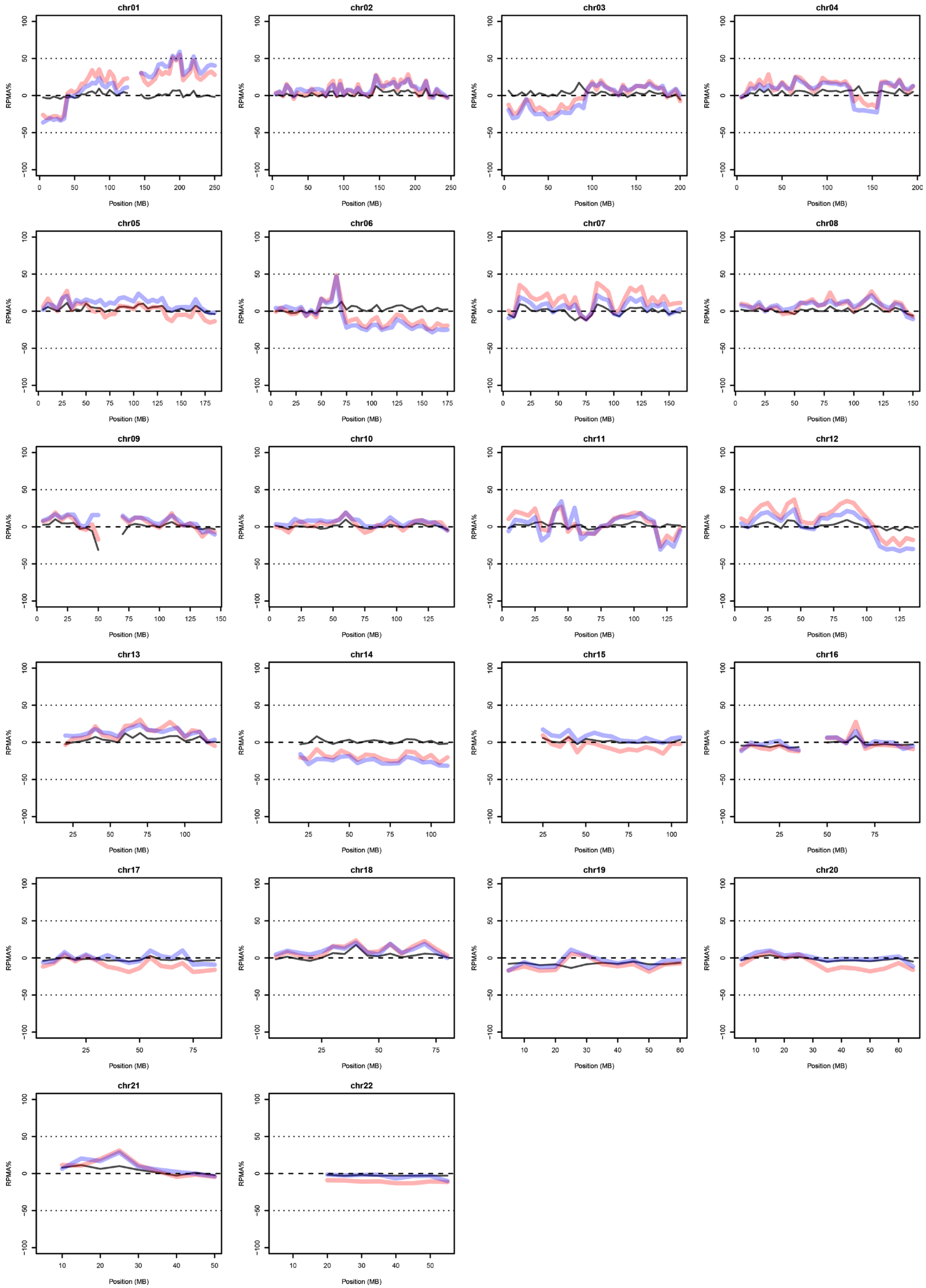

Pat4 Coverage deviation

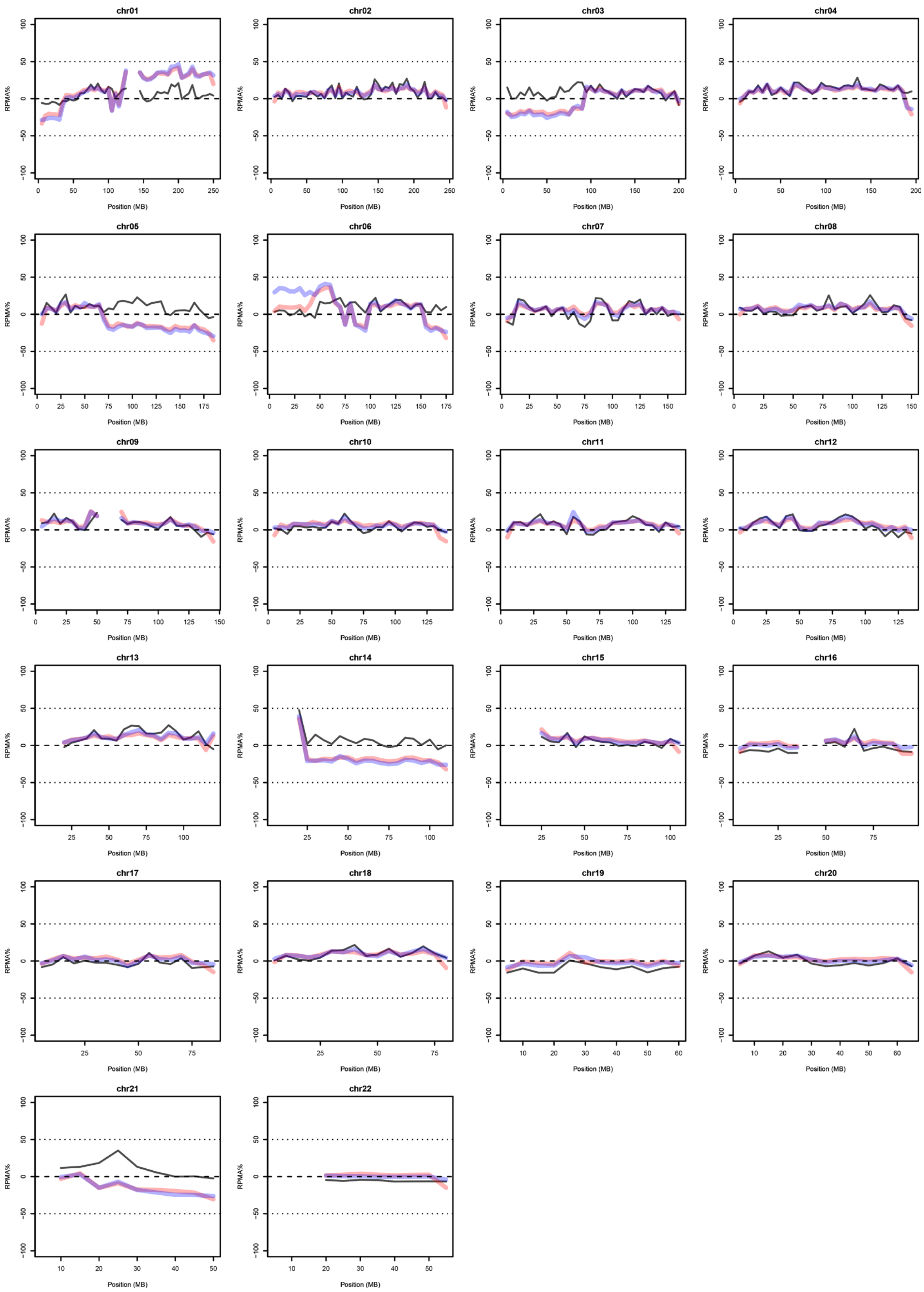

Supplementary Figure 5: Copy number variations (CNV) of patient 1–4. Of each patient CNV of non-tumor sample (black line), central tumor sample (red line) and peripheral sample (blue line) is shown. X-axis: chromosome length (megabases), y-axis: deviation from expected coverage, upper dotted line = +50%, lower dotted line = –50%.

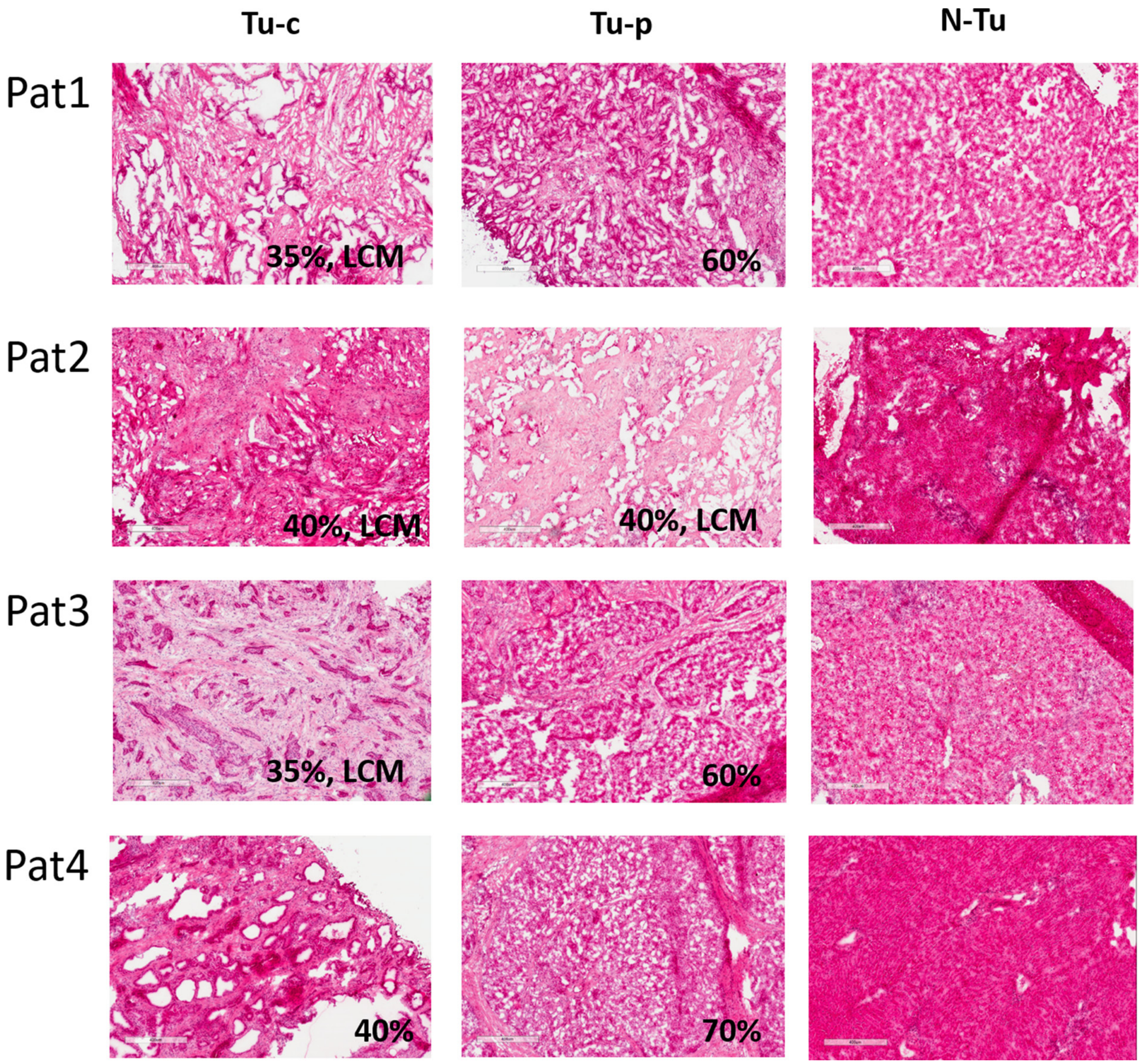

Supplementary Figure 6: Representative areas of frozen sections stained with hematoxylin and eosin (100x). LCM: in these cases, laser capture microdissection (LCM) was performed to remove parts with lower tumor content; percentage of tumor content is after LCM in these cases. N-Tu: Non-tumor sample, Tu-c: central tumor sample, Tu-p: peripheral tumor sample.

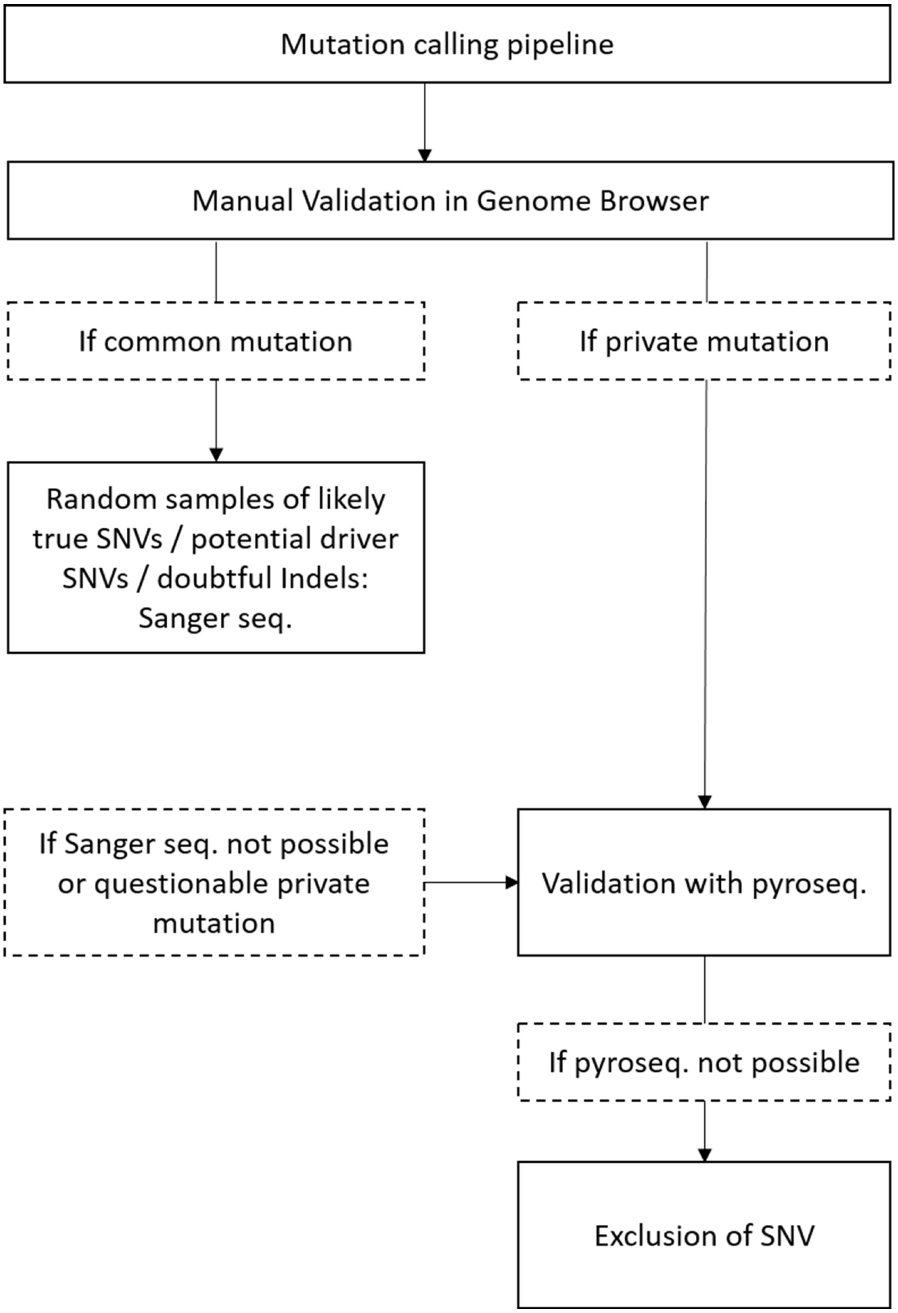

Supplementary Figure 7: Scheme of algorithm of validation of detected single nucleotide variants (SNV) in whole exome sequencing. Seq: sequencing.
