# Supplementary material for: Intratumoral heterogeneity of intrahepatic cholangiocarcinoma
Source: Oncotarget. 2017 Jan 27;8(9):14957–68. doi: 10.18632/oncotarget.14844 (PMC5362457; doi:10.18632/oncotarget.14844)
Supplement: Supplementary file 2 [file oncotarget-08-14957-s002.docx]

Supplementary Table S2: Details of all identified mutations

| Patient | Gene | Location | Variant | Category | Transcript | Amino acid change |
| --- | --- | --- | --- | --- | --- | --- |
| Pat1 | ADAMTS18 | Com | 16:77396033 A > G | syn | NM_199355 | = |
| Pat1 | AHDC1 | Com | 1:27876735 C > T | mis | NM_001029882 | Arg631Gln |
| Pat1 | ARR3 | Com | X:69489737 C > G | mis | NM_004312 | Asp28Glu |
| Pat1 | ASB10 | Com | 7:150873491 C > A | spl | XM_005249949 | NA |
| Pat1 | CD109 | Com | 6:74521929 T > G | mis | NM_133493 | Leu1235Arg |
| Pat1 | CDYL2 | Com | 16:80654740 G > A | syn | ENST00000562812 | = |
| Pat1 | CMYA5 | Com | 5:79034572 C > T | syn | NM_153610 | = |
| Pat1 | CNTRL | Com | 9:123900933 C > T | syn | NM_007018 | = |
| Pat1 | COBLL1 | Com | 2:165551735 G > C | mis | NM_014900 | Arg761Gly |
| Pat1 | DLGAP2 | Com | 8:1449559 C > T | mis | XM_005266038 | Pro68Leu |
| Pat1 | EGLN2 | Com | 19:41312522 C > T | syn | NM_080732 | = |
| Pat1 | FAM43A | Com | 3:194407699 C > T | syn | NM_153690 | = |
| Pat1 | FBXO21 | Com | 12:117604869 T > C | mis | NM_015002 | Ile343Val |
| Pat1 | GPAA1 | Com | 8:145138024 C > A | spl | NM_003801 | NA |
| Pat1 | GPR20 | Com | 8:142367311 C > T | mis | NM_005293 | Arg238Gln |
| Pat1 | GPRASP1 | Com | X:101911910 G > A | syn | NM_014710 | = |
| Pat1 | HES1 | Com | 3:193854763 C > T | mis | NM_005524 | Ser73Phe |
| Pat1 | LOXHD1 | Com | 18:44181276 G > A | syn | NM_144612 | = |
| Pat1 | LRRC16A | Com | 6:25466128 G > A | syn | NM_017640 | = |
| Pat1 | MAP10 | Com | 1:232942331 A > T | mis | NM_019090 | Asn521Ile |
| Pat1 | MSN | Com | X:64955135 G > A | mis | NM_002444 | Val268Ile |
| Pat1 | NFASC | Com | 1:204924009 C > T | syn | NM_001005388 | = |
| Pat1 | OR8S1 | Com | 12:48919484 C > T | mis | NM_001005203 | Arg24Trp |
| Pat1 | OSBPL3 | Com | 7:24849577 T > C | spl | NM_145322 | NA |
| Pat1 | PCDH8 | Com | 13:53418998 G > A | syn | NM_002590 | = |
| Pat1 | RIMS2 | Com | 8:105025753 G > A | mis | NM_014677 | Arg857His |
| Pat1 | RPAP1 | Com | 15:41829273 G > A | syn | NM_015540 | = |
| Pat1 | SEMA5B | Com | 3:122632056 G > A | syn | NM_001031702 | = |
| Pat1 | SPTBN5 | Com | 15:42145938 C > T | syn | NM_016642 | = |
| Pat1 | SSPO | Com | 7:149524996 C > T | mis | NM_198455 | Arg4975Trp |
| Pat1 | TCEB3 | Com | 1:24080930 C > T | mis | NM_003198 | Arg617Cys |
| Pat1 | TFAP2D | Com | 6:50712840 C > T | mis | NM_172238 | Arg302Trp |
| Pat1 | TRIM42 | Com | 3:140397360 C > T | mis | NM_152616 | Arg97Cys |
| Pat1 | TRMT2B | Com | X:100292961 G > A | mis | NM_001167970 | Thr128Met |
| Pat1 | TRPC5 | Com | X:111025292 C > T | syn | NM_012471 | = |
| Pat1 | TSEN54 | Tu-p | 17:73518368 C > T | syn | NM_207346 | = |
| Pat1 | UBE3B | Com | 12:109961835 G > C | mis | NM_130466 | Gly806Ala |
| Pat1 | ZHX1 | Tu-c | 8:124266147 G > A | syn | NM_007222 | = |
| Pat1 | ZNF613 | Com | 19:52448903 G > T | syn | NM_001031721 | = |
| Pat1 | RNF43 | Com | 17 :56435873 | non | NM_017763.4 | Gln422Ter |
| Pat1 | ABCC9 | Com | 12 :22015947 | mis | NM_020297.2 | Trp760Leu |
| Pat1* | CSMD1 | Com | 8:2824266 C > T | mis | ENST00000520002 | Val2977Met |
| Pat1* | CT47B1 | Tu-c | X:120009257 C > T | mis | NM_001145718 | Ala90Thr |
| Pat1* | DCAF4L2 | Com | 8:88885088 C > T | mis | NM_152418 | Arg371His |
| Pat1* | FAT2 | Com | 5:150924617 C > G | mis | NM_001447 | Arg2024Thr |
| Pat1* | GNPNAT1 | Tu-p | 14:53245128 C > G | mis | NM_198066 | Lys152Asn |
| Pat1* | IKBKAP | Com | 9:111670606 G > A | mis | NM_003640 | Pro480Leu |
| Pat1* | KCNE5 | Com | X:108868095 G > C | mis | NM_012282 | Thr52Ser |
| Pat1* | KDM4B | Com | 19:5135435 C > T | mis | NM_015015 | Thr724Ile |
| Pat1* | KIF2A | Com | 5:61658311 C > A | mis | NM_001098511 | Asn354Lys |
| Pat1* | KRAS | Com | 12:25398284 C > T | mis | NM_004985 | Gly12Asp |
| Pat1* | LIG4 | Com | 13:108863159 T > C | mis | NM_001098268 | Asp153Gly |
| Pat1* | MYT1L | Com | 2:1895999 C > T | mis | NM_015025 | Ser696Asn |
| Pat1* | NLRP8 | Com | 19:56477731 G > A | mis | NM_176811 | Arg789His |
| Pat1* | NRXN3 | Com | 14:79933641 C > T | mis | NM_001105250 | Arg109Trp |
| Pat1* | OR10J3 | Com | 1:159284161 T > G | mis | NM_001004467 | Ser97Arg |
| Pat1* | OTOF | Com | 2:26697512 C > T | mis | NM_194323 | Ala306Thr |
| Pat1* | SCN5A | Com | 3:38592173 C > T | mis | NM_198056 | Arg1897Gln |
| Pat1* | SMAD4 | Com | 18:48603147 G > C | mis | ENST00000593223 | Ser72Thr |
| Pat1* | SRC | Com | 20:36031737 Ins T | fra | NM_005417 | Thr524HisfsTer52 |
| Pat1* | ZNF207 | Com | 17:30687928 C > T | mis | NM_001098507 | Pro165Ser |
| Pat2 | ANKLE2 | Com | 12:133331504 C > A | mis | NM_015114 | Asp133Tyr |
| Pat2 | ATP11B | Com | 3:182605444 G > C | mis | NM_014616 | Ser929Thr |
| Pat2 | C21orf33 | Com | 21:45563112 G > A | mis | NM_004649 | Ala183Thr |
| Pat2 | C2orf71 | Com | 2:29296367 G > T | mis | NM_001029883 | Pro254His |
| Pat2 | CACNA1F | Com | X:49068712 A > T | syn | NM_005183 | = |
| Pat2 | CLGN | Tu-p | 4:141310426 T > C | syn | NM_004362 | = |
| Pat2 | CREM | Com | 10:35468173 T > C | syn | NM_183013 | = |
| Pat2 | DLG4 | Com | 17:7099860 T > C | mis | NM_001365 | Glu416Gly |
| Pat2 | EPX | Com | 17:56270742 C > T | mis | NM_000502 | Arg61Trp |
| Pat2 | HDAC9 | Com | 7:18767219 C > T | mis | NM_178423 | Thr580Met |
| Pat2 | HMCN2 | Com | 9:133234788 C > T | syn | XM_001726942 | = |
| Pat2 | HNRNPA1 | Com | 12:54675953 A > G | mis | NM_031157 | His120Arg |
| Pat2 | IGSF10 | Com | 3:151164153 G > A | non | NM_178822 | Gln1206Ter |
| Pat2 | MACF1 | Com | 1:39845931 T > A | mis | NM_012090 | Leu2450Met |
| Pat2 | MAGEB16 | Com | X:35820339 G > A | mis | NM_001099921 | Arg9Gln |
| Pat2 | MFSD11 | Com | 17:74735057 A > T | mis | NM_001242534 | His45Leu |
| Pat2 | MMADHC | Com | 2:150432342 C > G | syn | NM_015702 | = |
| Pat2 | NPAS4 | Com | 11:66190012 G > T | mis | NM_178864 | Ala140Ser |
| Pat2 | NXN | Com | 17:726880 C > T | mis | NM_022463 | Ala202Thr |
| Pat2 | OPN1LW | Com | X:153421993 C > T | syn | NM_020061 | = |
| Pat2 | OR2C1 | Com | 16:3406102 G > T | syn | NM_012368 | = |
| Pat2 | PCDHB1 | Com | 5:140431617 G > A | mis | NM_013340 | Gly188Arg |
| Pat2 | PRPF39 | Com | 14:45581559 A > T | mis | NM_017922 | Glu537Asp |
| Pat2 | RABGAP1L | Com | 1:174274135 A > T | mis | NM_014857 | Lys445Asn |
| Pat2 | RNF165 | Com | 18:44027572 C > T | mis | NM_152470 | Arg178Trp |
| Pat2 | SLC4A11 | Com | 20:3209805 G > C | mis | NM_001174090 | Leu695Val |
| Pat2 | SYT9 | Com | 11:7334808 T > A | mis | NM_175733 | Ile227Asn |
| Pat2 | TBC1D25 | Com | X:48399740 C > T | mis | NM_002536 | Pro48Leu |
| Pat2 | TROAP | Com | 12:49717771 G > A | syn | NM_005480 | = |
| Pat2 | PCDHB6 | Com | 5 :140531786 | mis | NM_018939.2 | Arg650Cys |
| Pat2* | BCL2L15 | Com | 1:114424474 C > T | mis | NM_001010922 | Gly132Glu |
| Pat2* | CD302 | Tu-c | 2\|2:160637386 DelTTAC | spl | NM_001198764 | NA |
| Pat2* | CNOT10 | Tu-c | 3:32776469 G > C | spl | NM_015442 | NA |
| Pat2* | DCAF4L2 | Tu-c | 8:88886051 C > T | mis | NM_152418 | Arg50His |
| Pat2* | DNAH11 | Com | 7:21640513 G > A | mis | NM_001277115 | Glu1074Lys |
| Pat2* | DOCK10 | Com | 2:225709490 C > G | mis | NM_014689 | Val850Leu |
| Pat2* | EPHA2 | Com | 1:16456809 G > A | mis | NM_004431 | Arg861Cys |
| Pat2* | FTSJ3 | Com | 17:61897271 Del GCA | del | NM_017647 | Val811_Arg812del |
| Pat2* | IDH2 | Com | 15:90631839 T > A | mis | NM_002168 | Arg172Trp |
| Pat2* | ITPR3 | Com | 6:33636917 G > A | mis | NM_002224 | Val725Met |
| Pat2* | KANSL1L | Com | 2:210962853 T > A | non | NM_152519 | Lys503Ter |
| Pat2* | LUC7L | Tu-p | 16:240067 G > A | mis | NM_018032 | Arg292Trp |
| Pat2* | MUC16 | Com | 19:9083609 G > A | mis | NM_024690 | Arg2736Trp |
| Pat2* | MUC2 | Tu-p | 11:1086402 C > A | non | NM_002457 | Cys1037Ter |
| Pat2* | TYMP | Tu-p | 22:50966074 T > G | mis | NM_001953 | Ile197Leu |
| Pat2* | ZNF638 | Tu-c | 2:71650547 T > G | syn | NM_014497 | = |
| Pat3 | ACTL10 | Com | 20:32255905 C > A | mis | NM_001024675 | Ala201Asp |
| Pat3 | ADAM8 | Com | 10:135086936 T > A | syn | NM_001164489 | Gln132Leu |
| Pat3 | C11orf65 | Com | 11:108277506 C > T | mis | NM_152587 | Arg138Lys |
| Pat3 | CKAP4 | Com | 12:106633077 T > A | mis | NM_006825 | Thr512Ser |
| Pat3 | FAM178B | Com | 2:97637976 G > A | mis | NM_001122646 | Arg76Cys |
| Pat3 | FBXL15 | Com | 10:104181724 C > T | mis | NM_024326 | Arg130Trp |
| Pat3 | FRYL | Com | 4:48537788 C > T | syn | NM_015030 | = |
| Pat3 | HECTD2 | Com | 10:93242776 G > A | mis | NM_182765 | Arg255Gln |
| Pat3 | IL36A | Com | 2:113764173 A > T | spl | NM_014440 | NA |
| Pat3 | IRF2BP1 | Com | 19:46388100 C > T | syn | NM_015649 | = |
| Pat3 | MCM7 | Com | 7:99698820 C > A | spl | ENST00000465688 | NA |
| Pat3 | MICALL2 | Com | 7:1477172 G > A | spl | NM_182924 | = |
| Pat3 | MTUS2 | Com | 13:29933455 C > A | mis | NM_001033602 | Pro998Thr |
| Pat3 | NAPRT | Com | 8:144657797 A > G | mis | NM_145201 | Tyr395His |
| Pat3 | NKG7 | Com | 19:51875750 G > T | mis | NM_005601 | Leu14Met |
| Pat3 | NKPD1 | Tu-p | 19:45662222 C > A | syn | NM_198478 | = |
| Pat3 | NPAP1 | Com | 15:24922982 G > T | syn | NM_018958 | = |
| Pat3 | OR13C5 | Com | 9:107361254 C > G | syn | NM_001004482 | = |
| Pat3 | PAPOLB | Com | 7:4901353 G > A | mis | NM_020144 | Ala30Val |
| Pat3 | PHF2 | Com | 9:96429395 A > G | mis | NM_005392 | Ile741Val |
| Pat3 | PIWIL4 | Com | 11:94318631 T > C | mis | NM_152431 | Met219Thr |
| Pat3 | PLEC | Com | 8:144995843 G > A | syn | NM_201382 | = |
| Pat3 | PPFIA2 | Com | 12:82070570 C > G | spl | NM_003625 | = |
| Pat3 | RANBP10 | Com | 16:67763669 C > T | mis | NM_020850 | Arg354Gln |
| Pat3 | S100A2 | Com | 1:153533968 G > A | mis | NM_005978 | Leu81Phe |
| Pat3 | TCF20 | Com | 22:42607292 T > A | mis | NM_181492 | Lys1340Asn |
| Pat3 | TTC34 | Tu-p | 1:2704142 C > T | syn | NM_001242672 | = |
| Pat3 | UHRF1BP1 | Com | 6:34838687 G > A | mis | NM_017754 | Gly1259Ser |
| Pat3 | ZFP62 | Tu-c | 5:180277928 C > A | syn | NM_001172638 | = |
| Pat3 | GPR173 | Com | X :53106312 | fra | NM_018969.5 | Glu171ArgfsTer47 |
| Pat3* | ABCC9 | Com | 12:22059071 G > T | mis | NM_020297 | Thr536Lys |
| Pat3* | ADSS | Com | 1:244600184 C > G | mis | NM_001126 | Val100Leu |
| Pat3* | BAP1 | Com | 3:52442489 C > A | spl | NM_004656 | NA |
| Pat3* | CHRDL1 | Com | X:109937424 T > A | mis | NM_001143982 | Ile248Phe |
| Pat3* | COMMD1 | Com | 2:62196170 C > G | mis | ENST00000445644 | Arg83Gly |
| Pat3* | EPHA2 | Com | 1:16474910 G > T | non | NM_004431 | Cys262Ter |
| Pat3* | GDF10 | Com | 10:48428952 G > A | mis | NM_004962 | Arg312Cys |
| Pat3* | GOLIM4 | Tu-p | 3:167745536 C > T | mis | NM_014498 | Asp535Asn |
| Pat3* | HDAC7 | Com | 12:48178569 A > C | mis | XM_005268967 | Leu953Arg |
| Pat3* | IDH1 | Com | 2:209113113 G > A | mis | NM_005896 | Arg132Cys |
| Pat3* | MUC16 | Com | 19:9085555 G > A | mis | NM_024690 | Thr2087Met |
| Pat3* | NEK7 | Com | 1:198266320 A > G | mis | NM_133494 | Ile250Val |
| Pat3* | SYNPO2 | Com | 4:119948208 C > G | mis | NM_133477 | Asp228Glu |
| Pat3* | TMEM217 | Com | 6:37180470 C > G | mis | XM_005248910 | Trp92Cys |
| Pat3* | UBR3 | Com | 2:170936486 C > A | mis | NM_172070 | Leu1788Met |
| Pat3* | ZNF235 | Com | 19:44791537 G > A | mis | NM_004234 | Thr684Met |
| Pat4 | ADCY7 | Com | 16:50324552 C > T | mis | NM_001114 | Ala119Val |
| Pat4 | ANKFN1 | Com | 17:54559803 C > T | syn | NM_153228 | = |
| Pat4 | ANKS6 | Com | 9:101542615 C > G | syn | NM_173551 | = |
| Pat4 | ARL2 | Com | 11:64786290 G > C | mis | XM_005273992 | Gly142Arg |
| Pat4 | CACNG8 | Com | 19:54483177 G > A | mis | NM_031895 | Gly142Arg |
| Pat4 | CD46 | Com | 1:207934783 A > G | mis | NM_002389 | Glu222Gly |
| Pat4 | CXCR4 | Tu-c | 2:136872724 C > A | syn | NM_001008540 | = |
| Pat4 | DCC | Com | 18:50683801 G > A | mis | NM_005215 | Arg446His |
| Pat4 | FLG | Com | 1:152281947 C > T | syn | NM_002016 | = |
| Pat4 | FPR2 | Com | 19:52272706 C > T | syn | NM_001005738 | = |
| Pat4 | HORMAD1 | Com | 1:150680764 C > G | mis | NM_032132 | Cys172Ser |
| Pat4 | IARS | Com | 9:95003238 C > T | syn | NM_002161 | = |
| Pat4 | ITGAL | Com | 16:30495161 C > T | mis | NM_002209 | Arg246Trp |
| Pat4 | KRT31 | Com | 17:39552699 C > A | syn | NM_002277 | = |
| Pat4 | KRTAP4-11 | Com | 17:39274415 C > T | syn | NM_033059 | = |
| Pat4 | KTN1 | Com | 14:56168064 A > G | mis | ENST00000554294 | Thr129Ala |
| Pat4 | L3MBTL2 | Com | 22:41621051 C > T | syn | NM_031488 | = |
| Pat4 | LOC729159 | Com | 16:60393307 C > A | syn | XM_001129515 | = |
| Pat4 | MSMB | Com | 10:51556821 C > T | syn | NM_002443 | = |
| Pat4 | NEURL4 | Com | 17:7224433 C > T | mis | NM_032442 | Gly1120Arg |
| Pat4 | OR52N1 | Tu-c | 11:5809201 G > T | syn | NM_001001913 | = |
| Pat4 | OR5H8 | Com | 3:98030983 C > T | mis | ENST00000394191 | Ser76Leu |
| Pat4 | PCDHGA 12 | Com | 5:140812501 G > T | syn | NM_003735 | = |
| Pat4 | PTPRC | Com | 1:198682129 C > A | mis | NM_002838 | Gln407Lys |
| Pat4 | SALL3 | Com | 18:76753758 C > T | syn | NM_171999 | = |
| Pat4 | SDF2L1 | Com | 22:21997271 C > T | mis | NM_022044 | Ala103Val |
| Pat4 | SYNGR1 | Com | 22:39777894 A > T | mis | NM_004711 | Gln226Leu |
| Pat4 | TENM1 | Com | X:123554423 T > G | mis | NM_014253 | Asn1567His |
| Pat4 | USP54 | Com | 10:75276724 T > G | mis | NM_152586 | Ser1154Arg |
| Pat4 | FNIP1 | Tu-p | 5 :131132613 | i_c_v | NM_133372.2 | p.Met1 |
| Pat4 | NUP88 | Com | 17 :5290963 | fra | NM_002532.4 | Lys600GlufsTer21 |
| Pat4* | AP1M2 | Com | 19:10692245 T > C | mis | NM_005498 | Asn155Ser |
| Pat4* | BAP1 | Com | 3:52441456 Ins C | fra | NM_004656 | Asn133GlnfsTer10 |
| Pat4* | C11orf65 | Com | 11:108205939 A > G | spl | XM_005271415 | NA\|NA |
| Pat4* | C18orf8 | Tu-p | 18:21089203 A > C | mis | NM_013326 | Glu123Ala |
| Pat4* | CNKSR3 | Com | 6:154831240 G > T | syn | NM_173515 | = |
| Pat4* | DNAH9 | Com | 17:11872774 T > C | mis | NM_001372 | Phe4464Ser |
| Pat4* | EDN3 | Com | 20:57899485 C > T | mis | NM_207034 | Arg230Cys |
| Pat4* | FLG2 | Com | 1:152323460 A > T | mis | NM_001014342 | Ser2268Thr |
| Pat4* | GNA14 | Tu-p | 9:80143977 C > T | spl | NM_004297 | NA |
| Pat4* | MSH6 | Tu-p | 2:48010469 C > T | mis | NM_000179 | Arg33Cys |
| Pat4* | NIT1 | Tu-p | 1:161089141 G > T | mis | NM_001185092 | Gly106Cys |
| Pat4* | OIP5 | Com | 15:41605472 T > G | mis | NM_007280 | Glu198Ala |
| Pat4* | OR52E8 | Tu-p | 11:5878152 A > T | mis | NM_001005168 | Phe261Ile |
| Pat4* | OR5D14 | Tu-p | 11:55563334 G > T | mis | NM_001004735 | Met101Ile |
| Pat4* | PLA2R1 | Tu-p | 2:160879191 T > C | mis | NM_001195641 | Thr427Ala |
| Pat4* | PPFIA4 | Tu-p | 1:203013866 G > T | mis | ENST00000367240 | Ala207Ser |
| Pat4* | PRKAB1 | Tu-c | 12:120106054 G > C | mis | NM_006253 | Gly2Ala |
| Pat4* | PRTG | Tu-c | 15:55933341 C > A | mis | NM_173814 | Asp703Tyr |
| Pat4* | PTPDC1 | Tu-p | 9:96859884 G > C | mis | NM_177995 | Val292Leu |
| Pat4* | RFX8 | Tu-p | 2:102038162 C > G | spl | XR_244895 | NA |
| Pat4* | SALL1 | Com | 16:51172703 A > G | mis | NM_002968 | Ser1144Pro |
| Pat4* | SETD1A | Tu-p | 16:30977357 C > T | mis | NM_014712 | Pro719Ser |
| Pat4* | UBALD1 | Tu-p | 16:4660515 G > T | mis | NM_145253 | Tyr54Ter |
| Pat4* | ZC3H14 | Tu-p | 14:89068289 T > G | mis | NM_024824 | Met459Arg |
|  |  |  |  |  |  |  |

## Supplementary Table S2: Details of all identified mutations

Suppl. Table S2: All detected common (com) and private mutations in either central (Tu-c) or peripheral (Tu-p) tumor sample. Transcript ID is given from Reference Sequence (RefSeq). If RefSeq ID was not available, Ensemble ID is provided. Mis: Missense mutation, spl: splice site, fra: frameshift, syn: synonymous, non: nonsense, del: deletion, i_c_v: initiator codon variant. Asterisk is indicating validated mutations.

## Supplementary Table S4: Primer sequences (pyrosequencing)

| Patient | Gene | Forward Primer  (5’>3’) | Reverse Primer  (5’>3’) | Sequencing Primer  (5’>3’) |
| --- | --- | --- | --- | --- |
| Pat1 | KIF2A | AGCTCGAGATGTCTTTTTAATGC* | GTTCCCACTTACCTTTCCACTATA | TTGAAGTTCTAGCTTCTTAT |
| Pat1 | GNPNAT1 | CCTACCCCCTTTTTAAATTTCAG | CCGACACATGTAGTTTTCTTCAGA* | GCAAGAAACTGAACTGTTA |
| Pat1 | MYT1L | TCATCCCTGTCCCTGTCTCC | CCTCCATGTCGTGCGTGTAGT* | GCAGCTACGCGCCCA |
| Pat1 | FAT2 | ATGGTCCAGTCAGCAGGTGTGT | CCTCACTTCCACTGCCAACTC* | AGCAGGTGTGTTGCAGA |
| Pat1 | SMAD4 | TTGCGTCAGTGTCATCGACAG | AGAATGCAAACAGGGTCATAGGC* | GGAATAGCTCCAGCTATC |
| Pat1 | CT47B1 | CGACAGCAGCGACATGGT* | CCACCGCCAAGTCGAAGTT | CTCCTCTTCCTCCGT |
| Pat1 | KDM4B | GCTGCCCGGCCACATTAC* | GTCGTCGCCGATGTAGGAGTT | TCAGGGATGAGCGGT |
| Pat1 | NRXN3 | CCAATGACAGGCCCAGCA* | ATGCGGACCAAGATGCCA | GGCAAGGCGGTCAGA |
| Pat1 | IKBKAP | TGTGGGTGGAAGTGGATTTAA* | CTGTTCCCAGAAGCCCAAGATA | TGTATCTCTTTTCCAAATG |
| Pat1 | LIG4 | TTTGTGTTGAAGCCAAGATGTT* | ATCATCCGTATAAGCCACTTTTG | AATTATTGCTGGCAATT |
| Pat1 | OR10J3 | CATCATTCCCCATATGCTTTC* | TGTTGATGCCAAAGGTGAGAT | GAGCTGAGTGGCACA |
| Pat1 | KCNE5 | CTGCACCACCGGGGTAAT | TACGGGAGCGGGTGTAGG* | GTGGGCCGCGAGGTG |
| Pat1 | NLRP8 | ATGCAGGGTGCTGAGATCC | CACTCTAACCCCCCTCTCAAAT* | TGAGATCCCCCCGGT |
| Pat1 | FNIP1 | TAG GAG CAG GGG CCT AGC AA* | AGA GCT TCT GGA ACA GCG TAG G | GAA CAG CGT AGG GGC |
| Pat2 | EPHA2 | CTACCAGCTCATGATGCAGTG* | TTGTCCAGGATGCTGACGA | TCAGCGAACTTGGGG |
| Pat2 | IDH2 | CATCCCACGCCTAGTCCCT | CCTGGCCTACCTGGTCGC* | AGCCCATCACCATTG |
| Pat2 | DOCK10 | TGGGCTTAAGGGATTAGGACATTC | ATGGTGGCAAACCACTTTTCAA* | CACCTGAGTATTTACTGTTG |
| Pat2 | MUC2 | CCACCTGCCCAGATGTGAG* | GGATGCTGCACTGCTTCTCG | GGTGCGGGTTCAGGC |
| Pat2 | FTSJ3 | ACCACAAAGGTACACAGGGCTAAA* | TTTGGTCCTTCTTCATCCTTGAGT | GACTCCAGCTGGCCG |
| Pat2 | LUC7L | GTGGGGCCCTCATAGTGTT | GTGGCGCCGATGTCTATC* | GAGCGACGGAAATTG |
| Pat2 | TYMP | GTATCGTGGGTCAGAGTGAGCA | GTCCACGGTGGCTGTCACA* | TGGTTCCTGCGGACG |
| Pat2 | MUC16 | TTCTTCCACTTCCCCCTCTAA | GAAAGTGCCACTGTGATCGA* | CCCTCTAAGATTGTTGCTC |
| Pat2 | DCAF4L2 | AAGACAGTCAGAGTGGGACTCAAT* | TATGCGGTTAAATCGGTCG | TGCATGCAGCTTACA |
| Pat2 | CNOT10 | AGCAGCGAAAGCAGTGAAAC* | TTTTGCCAAAGGAGGTCACA | GAGGTCACAAGGATTAGAA |
| Pat2 | CD302 | GGCATGTTTTATGACACAGATGGT | TGAAAAGCCATCAGTTTTGCTACA* | TGTTTTATGACACAGATGGT |
| Pat3 | GOLIM4 | ATAGACATGAGCCTCGTGAACAAG | TCAACTCTCTTTCCCACCTTATGA* | AAGGACCCCGAGAAG |
| Pat3 | ZNF235 | AGTTGGAGTGCTGGTCTCAGTG | GAACCACACCTCTCATTTCTACAG* | ACAGGAGAGAAACCCTAT |
| Pat3 | HDAC7 | ACATGGCTGATTCCTCTTACGG | GGAACCATGTCCTGGAATCTG* | GGTGGGTGCCCTCACA |
| Pat3 | ABCC9 | GCCTGGGAACACATTTTCTG* | GGTTATTCTGGTTGCCCTTAAA | GCAACTTACTGGAGAGTGAT |
| Pat3 | BAP1 | GTCTCTACCTTGGTGGATGATACG | TCCACTTCCCAAGCAAAAACAT* | TGTTCTTTGCCCACC |
| Pat3 | ADSS | CGTTGGGATTTGTTGGTTTT* | GCTTCTTCAAACAATCCAGGTAG | CCTTTAGGAAATGGTGTG |
| Pat3 | SYNPO2 | AACTGTCCCTTTCACAGGAGAGA | AGGGTCTGCTTTCTCATTAGTAGG* | CTGAAAAATCTAAGTCTCCT |
| Pat3 | COMMD1 | GGGTATTCATAGGCACAATCATGG | GAGAATCACTTGAGCCCAGGAGTT* | GGAGTTCAAGGCCAC |
| Pat4 | DNAH9 | TGCCGCAGTGTCTATTCCT | GCAGGATGCTAAATCTGGAGAAG* | CACCTACGTGTGGACTT |
| Pat4 | FLG2 | GGCCACAGTGAGTCCAGTGAC | GGCCCTGAATGTGTCCTGAA* | AAGTGCACTCATGGGG |
| Pat4 | CNKSR3 | GGCGAAGCGGTTTACCAT* | CTAGTCCAGTCCACCACTTGTTTG | GGCTCCACTTGGTCAC |
| Pat4 | ZC3H14 | TTGGCTCAGATTACTATGACATGG* | TTTGGTGCCACTACTACTTCCTC | GTGTCTGCATGGACC |
| Pat4 | EDN3 | TCGCCCTCGCTCCATCTA* | TGTCCTCCTAAGGGGCTCC | CCTGAAAGAGGCAGC |
| Pat4 | OR5D14 | TGAGAACTTGGTAATGGCAGATAA | GCCAGCAAGAAAGACTCTGTCA* | TTCTACTTTAGCTGCATGA |
| Pat4 | PLA2R1 | CGTTCTTGTCAGGCTGATAACAGT | AAAGGCAGCTGGGACTCA* | AGAGGTGGAGTTTCTTGTA |
| Pat4 | MSH6 | GTCTCCGGCGCTGAGTGA | CCGCCTGGGGAAGGAGAG* | TCACGCGAAGGCGGC |
| Pat4 | SETD1A | CTCGGCTCCATCAGCTGC | CTCCTGCCCCTGTGCATA* | GCCTTCCTCCCGTTT |
| Pat4 | RFX8 | CGAAATGTGAGGAACTGAGAACT | TGACGGGGTCTCTTCAAATAAA* | TGAGGAACTGAGAACTTGA |
| Pat4 | PRKAB1 | GTTCCGGGAGTCCCTTGCT | CGCGCTCACTGCTGGTATT* | CGCAGACCCCCATCA |
| Pat4 | PRTG | CAACATAGACGATGGCTATCAGG | ACTTACACACGCATCCTGGAGT* | CGATGGCTATCAGGCA |
| Pat4 | C18orf8 | GCCAACATTCTAGGATTCTGCT* | GGTAAAATTCGATTCCTTGATCTG | TTGATCTGTTATGAAGACAA |
| Pat4 | GNA14 | GCGATGGACACGCTAAGGATAC* | TTCACAAATAGGGCAAGCACTCC | GGGCAAGCACTCCCT |
| Pat4 | NIT1 | ACCCTGCAGAGACGCTACACCT* | TCCCTCGCTATTTCCCTGATACCT | TCCAAAAGTTTCCCAC |
| Pat4 | OIP5 | AAGCCATAGTAAATGCATCAGAGA* | CATTTCATCATCCCCAAGTTCA | CCATCATGAAATTTTACC |
| Pat4 | PPFIA4 | GGAAGGGTCTGGAATGTATGGG | CTTCCACAGGCGTTTCGG* | GTGCGGGATGGAGCG |
| Pat4 | PTPDC1 | ATCTAATTCGCCAGCGTCA | GGCCTGTTCTCCGCTAAGT* | GCCAAAAATTATCCACC |
| Pat4 | OR52E8 | CTCAACACCTGTGGTTCTCATATT | GGGATATTATGGCCAAAACGA* | AGCCTTTTTTACACCAG |
| Pat4 | UBALD1 | CAGCCCTCAGCGCCTTTTT | GGGACTCCGGGGACACTTAC* | AGACCAACATCCCCTA |

Suppl. Table S4: Primer sequences of genes validated with pyrosequencing. Asterisk is indicating the biotinylated primer.
